# Lignin-Containing Cellulose Acetate Films from Grapevine Waste: A Sustainable Path to Compostable Bioplastics

**DOI:** 10.1021/acssuschemeng.5c07998

**Published:** 2025-09-16

**Authors:** Raffaella Lettieri, Alice Caravella, Giulia Quintarelli, Cadia D’Ottavi, Silvia Licoccia, Emanuela Gatto

**Affiliations:** † Department of Chemical Science and Technologies, 9318 University of Rome Tor Vergata, via della Ricerca Scientifica 1, Rome 00133, Italy; ‡ Splastica srl, spinoff of the University of Rome Tor Vergata, Via della Ricerca Scientifica 1, 00133 Rome, Italy

**Keywords:** lignocellulosic biomass, green pretreatments, natural polymers, biodegradable films, circular economy

## Abstract

Grapevine shoots, a viticultural residue rich in cellulose (∼34%) and lignin (20–27%), were valorized through an eco-designed biorefinery process integrating autoclave-assisted pretreatment and organosolv extraction. This approach enabled the corecovery of cellulose and lignin under mild, sulfur-free conditions, followed by a chlorine-free bleaching that retained ∼7 wt % lignin within the cellulose. Rather than being removed, lignin was preserved as a natural additive for its antioxidant and UV-shielding functions, reducing the need for synthetic components. The lignin-containing cellulose was acetylated (DS ≈ 1.7) and plasticized with glycerol to produce biodegradable films. These films fully disintegrated under home composting within 8 weeks, outperforming commercial cellulose acetate. The process aligns with green chemistry principles and EU circular economy goals, offering a sustainable route to multifunctional bioplastics from agricultural waste.

## Graphical abstract

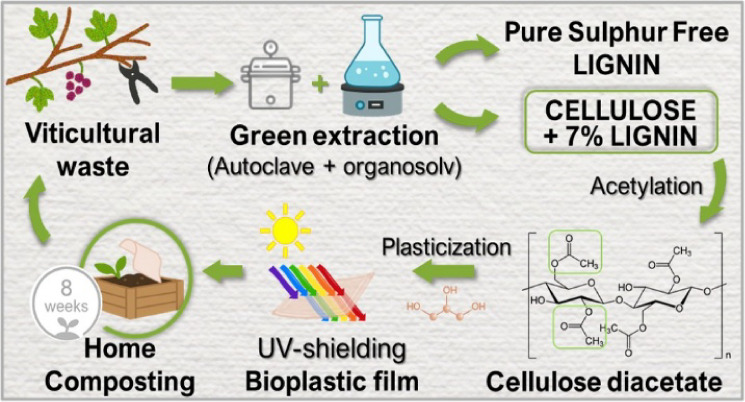

## 1 Introduction

The global environmental crisis resulting from the excessive accumulation of both plastic and organic waste calls for a fundamental shift in how we design materials and manage resources. With over 400 million tons of plastic produced annually^1^ and billions of tons of organic waste generated worldwide,^2^ the current linear economic model, based on extraction, use, and disposal, is proving environmentally and economically unsustainable. In response, the circular economy model promotes waste minimization, keeping materials in use for as long as possible. The recent UN Environment Programme (UNEP) calls for a shift “beyond the age of waste” by transforming waste into valuable secondary raw materials and reducing dependence on virgin resources, promoting the conversion of organic waste into new feedstocks.^3,4^ In this context, lignocellulosic biomass emerges as a strategic renewable resource, rich in natural polymers, such as cellulose and lignin, which can be recovered and reintroduced into sustainable value chains.

However, many currently available biobased materials fail to fully exploit the potential of natural polymers. They often consist of limited amounts of biomass embedded in fossil-based or nonbiodegradable matrices, compromising their recyclability and environmental compatibility.^5–8^ The use of natural polymers in biomaterials is also limited because they frequently lack the thermoplastic properties required for industrial scalability.^9–11^ Moreover, industrial processes concerning lignocellulosic biomass often target cellulose as the main valuable product, while lignin is discarded or underutilized, mainly due to its structural complexity, despite its chemical richness and functionality.^12–14^ However, recent research has been shifting this perspective. For example, Yao et al. demonstrated that microwave-assisted organosolv extraction enables the recovery of structurally preserved lignin, which, when incorporated even at low concentrations, enhances the mechanical properties of biobased resins.^15^ At a broader level, Tardy et al. highlighted the potential of lignin as a key functional component in sustainable material systems designed within circular economy frameworks.^16^ These findings emphasize the untapped potential of lignin not merely as a byproduct but as a multifunctional additive that can improve the performance and sustainability of biopolymers. To overcome these limitations, we propose a holistic and eco-designed approach to the recovery and transformation of natural polymers from agricultural residues into high-performance, fully bioderived materials. A key innovation in our strategy is the intentional preservation of a controlled amount of lignin within extracted cellulose. Rather than removing lignin entirely, as is typical in many purification protocols, we retain it as a functional component that enhances the antioxidant and UV-shielding properties of the final product.^17,18^ This eliminates the need for synthetic additives and further aligns the material with circular design principles.

Lignocellulosic biomass derived from agricultural sources such as wheat, maize, rice, and sugarcane represents a cost-effective and abundant feedstock for the production of energy, chemicals, and bioplastics.^19–22^ These residues typically contain 40–60% cellulose, 20–40% hemicellulose, and 10–25% lignin.^23–25^ Viticulture, in particular, generates significant amounts of underused lignocellulosic waste, including grapevine shoots, produced at rates of 1.4–2 tons per hectare, which are rich in cellulose (∼34%) and lignin (20–27%).^26^ Italy, accounting for over 30% of European wine production, offers substantial potential for the valorization of this biomass.^27^

In the present work, grapevine shoots were selected as model lignocellulosic feedstocks to demonstrate a fully integrated, low-impact recovery process. Starting from powdered biomass, we evaluated the efficacy of three pretreatment techniques emerged as effective and environmentally advantageous options,^28^ autoclave-assisted extraction (AAE), ultrasound-assisted extraction (UAE), and microwave-assisted extraction (MAE), with the aim of disrupting the complex biomass matrix while minimizing chemical input and energy use.^29^ Among these, AAE proved to be the most effective in facilitating polymer separation and reducing lignin–polysaccharide contamination. The pretreated biomass was then subjected to organosolv extraction, which delignifies the biomass under mild and sulfur-free conditions,^30^ yielding a cellulose-rich solid and a lignin fraction that, being sulfur-free, has been successfully used for drug-delivery pharmaceutical applications.^31^ Organosolv lignins exhibit enhanced phenolic functionality due to the cleavage of aryl–ether bonds during extraction. The use of organic solvents significantly limits condensation reactions, resulting in a structure that is more closely aligned with native lignin, more homogeneous in structure, and has lower molecular weight compared to lignins derived from other industrial processes, such as Kraft, soda, or bisulfite pulping.^32,33^ Recently, it has been reported that pilot-scale implementations demonstrated the feasibility of organosolv extraction also at an industrial scale.^30^

An optimized, chlorine-free bleaching process was developed to remove hemicellulose and reduce lignin content, while deliberately retaining ∼7 wt % of residual lignin in the cellulose. To impart to cellulose thermoplastic properties, it was acetylated to obtain cellulose acetate, which was plasticized with glycerol and cast into a cellulose-diacetate-based Bioplastic film, designed to be fully compostable. Unlike conventional approaches aimed at producing pure cellulose or isolated lignin, this strategy allows the development of materials with intrinsic UV-shielding and antioxidant properties, resulting from residual lignin. To our knowledge, this represents a unique valorization pathway for grapevine pruning waste, integrating mild, energy-efficient extraction, controlled lignin retention, polymer acetylation optimized to yield cellulose acetate with a tunable degree of substitution (balancing thermoplasticity and biodegradability), and eco-conscious, solvent- and additive-minimized film processing. The resulting Bioplastic meets key sustainability criteria outlined in Regulation (EU) 2025/40 on packaging and packaging waste, including compostability, compatibility with organic waste collection systems, and contribution to waste reduction and circular economy objectives (Articles 6, 7; Recitals 2, 15, 53).^34^ The entire project was conceived in line with the “waste hierarchy” principle of the Waste Framework Directive, emphasizing waste prevention and the design of biodegradable alternatives to conventional plastics.^35^ Moreover, the synthesis of the Bioplastic was guided by the Twelve Principles of Green Chemistry proposed by Anastas and Warner,^36^ adopting a quali-quantitative approach aimed at: (i) minimizing overall waste; (ii) fully valorizing all biomass fractions through selective separation; (iii) employing safer solvents and reducing hazardous reagents; (iv) utilizing renewable, second- and third-generation lignocellulosic feedstocks; and (v) designing materials intended for rapid degradation in soil, thereby minimizing environmental persistence in case of mismanagement.

## 2 Materials and Methods

*Biomass* Lignocellulosic biomass was obtained from pruning residues of *Vitis vinifera* collected in September 2021 from a vineyard located in southern Italy (Ordona, Foggia; coordinates: 41°18′54″N, 15°37′52″E). The biomass mixture included grape seeds, stalks, and vine shoots. The shoots were manually selected, thoroughly washed with distilled water, ground with a mechanical grinder, sieved through a 0.5 mm mesh to obtain a uniform powder, and oven-dried at 60 °C overnight.

*Reagents and Solvents* Ethanol (≥99.5%), sodium hydroxide (NaOH), hydrogen peroxide (30%), glacial acetic acid (≥99.8%), acetic anhydride, glycerol (≥99.5%), and acetone (≥99.5%) were supplied by Sigma-Aldrich. Cellulose acetate (manufacturer-provided specifications: Mn ∼ 30,000 by GPC, acetyl content 39.3–40.3 wt %), α cellulose, and lignin were obtained by Merck Life Science. Hydrochloric acid (37%) and sulfuric acid (95–97%) were purchased from Carlo Erba Reagents. Ultrapure Milli-Q water (resistivity 18.2 MΩ·cm) was used throughout.

*Pretreatments* For microwave-assisted extraction (MAE) and ultrasound-assisted extraction (UAE) pretreatments, a sample of dry powdered biomass in ethanol: water (60/40, v/v) with a liquor-to-solid ratio of 20:1 (mL/g) was used. Microwave-assisted extraction (MAE) was performed by using a CANDY (model CMW20SMW) microwave system operating at a fixed frequency of 2450 MHz. The sample was irradiated at 420 W for 80 s. Although the maximum output power of the microwave is 700 W, the applied power was set to 420 W. The temperature was not continuously monitored; however, the short exposure time and moderate power were chosen to minimize thermal degradation while effectively disrupting the biomass matrix. UAE pretreatment was performed using an Elmasonic ONE ultrasonicator, Elma Schmidbauer GmbH, Germany, for 1, 2, and 3 h at 35 kHz, at room temperature. Autoclave-assisted extraction (AAE) was carried out using the same liquor-to-solid ratio, but only distilled MQ water was used. The mixture was placed in an autoclave bottle at 121 °C and 1 atm for 30 min. All pretreatments were performed in duplicate. The pretreatment parameters were optimized by adapting protocols reported in the literature^37–40^ to grapevine shoots. Sonication was carried out at different durations (1–3 h) in order to evaluate the effect of treatment time. Microwave pretreatment was applied at 480 W for 80 s, while autoclave pretreatment was performed under controlled time, temperature, and pressure conditions to generate a vapor–thermal environment.

*Extraction procedure* After AAE pretreatment, all wet solid biomass was separated from the liquid phase by filtration and put in a round-bottomed flask together with ethanol: water (60/40, v/v) and 5 drops of 0.1% HCl per gram of biomass were added as a catalyst. For MAE and UAE pretreatment, the same amount of catalyst was added directly. The liquid-to-solid ratio was 20:1 (mL/g) based on the dry weight of the biomass before pretreatment. According to Xu et al.,^40^ the extraction was carried out at 85 °C for 4 h under stirring and refluxing. After filtration, the solid part (containing cellulose) was washed twice with acidified hot water and then with only hot water and allowed to dry at room temperature. Three volumes of ethanol were added to the liquid part, and it was allowed to stay overnight at room temperature to precipitate hemicellulose. Then, hemicellulose was separated by filtration using a vacuum pump and the solid acid-insoluble lignin was obtained from the liquid solution by the addition of acidified water (pH ∼ 2) after evaporation of the organic solvent and then freeze-dried (Figure 1).

**Figure 1:**
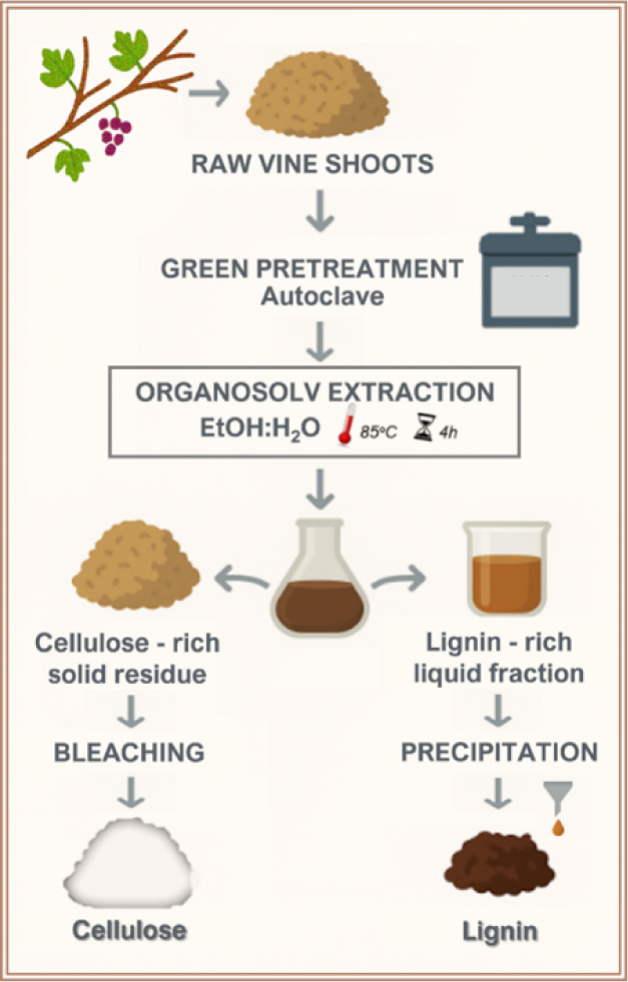
Scheme describing waste biomass pretreatment and extraction procedures.

*Bleaching* To obtain bleached cellulose, different bleaching treatments were tested. Initially, bleaching was carried out following the procedure described by Gabriel et al., 2020 (Procedure A),^41^ which involves treatment with 7.5% H_2_O_2_ and NaOH under stirring for 30 min at 100 °C. To optimize the process from a sustainability perspective, two NaOH concentrations (4% and 10%) were tested, corresponding to protocols A-1 and A-2. By combining the experimental conditions described by Gabriel et al.^41^ with those reported by Tezcan and Atici, (10% and 12% NaOH, 6 h at room temperature)^42^ further optimization was pursued developing two more bleaching procedures (procedures B-1 and B-2, where 10% and 12% NaOH were used, respectively). Procedures A-1 and A-2 reproduce the original protocol of Gabriel et al., while procedures B-1 and B-2 represent hybrid approaches that integrate conditions from both studies. Detailed bleaching conditions are reported in Table 1. After bleaching, the mixtures were thoroughly washed with hot water and dried in an oven at 60 °C overnight until a constant weight. All experiments were carried out in duplicate.

**Table 1:** Procedural Description of the Bleaching Treatment and Its Optimization

Bleaching procedure	NaOH conc.	H_2_O_2_ conc.	*t* (min)	*T* (°C)	ref.
A-1	4%	7.5%	30	100	^41^
A-2	10%	7.5%	30	100	^41^
B-1	10%	7.5%	60	100	^41,42^
B-2	12%	7.5%	60	100	^41,42^

*Calibration Curve for Lignin Quantification in the Cellulose Sample* Five standard samples were prepared by blending pure cellulose with pure lignin at known weight ratios of lignin (0%, 5%, 15%, 25%, and 40%). Each mixture was finely ground and homogenized to ensure representative sampling. FTIR-ATR spectra were recorded for each sample in the range of 4000–600 cm^–1^ (three replicates per sample) after applying baseline correction. Specific IR absorption bands were selected for analysis: the lignin band at ∼1510 cm^–1^ (C=C stretching of aromatic rings) and the cellulose band at 1030 cm^–1^ (C–O stretching in glycosidic ether bonds) with minimal spectral overlap. The lignin index was calculated as the ratio of the absorbance at 1515 cm^–1^ to that at 1030 cm^–1^. This index was plotted against the known lignin content of each standard, and a linear regression was performed to obtain the calibration curve. The lignin content in the unknown sample was then estimated by interpolating its corresponding lignin index into the regression equation. Prior to FTIR-ATR analysis, the unknown extracted cellulose samples were finely ground to ensure a homogeneous distribution of residual lignin throughout the powder. Spectra were recorded in triplicate at different positions of each sample to account for any local heterogeneity.

*Acetylation of cellulose* The acetylation of bleached cellulose was performed using a mixture composed of 18 mL of glacial acetic acid (as a solvent) and 5 mL of acetic anhydride per gram of cellulose. Three drops of sulfuric acid were added as a catalyst for each gram of cellulose. The reaction was carried out in a round-bottom flask. This protocol was developed by integrating elements from previously reported procedures.^43,45^ In the first referenced method, sodium bisulfate was used to reduce the required amount of sulfuric acid, while the second employed sulfuric acid alone as the catalyst. Based on stoichiometric calculations, the amounts of cellulose and acetic anhydride were aligned with those described by Djuned et al.,^43^ allowing for a reduction in the total catalyst amount by half. Sodium bisulfate was omitted, and overall reagent use was minimized to enhance the sustainability of the process. The resulting cellulose acetate (CA) was precipitated by the addition of an excess of distilled water and stirred at room temperature for 30 min. The precipitate was then filtered and washed thoroughly with distilled water until the filtrate reached pH 6. The purified CA was dried at room temperature, ground, and sieved by using a 177 μm mesh.

*Determination of degree of acetylation* The degree of acetylation (DA) of the cellulose acetate obtained from the extracted cellulose was determined following two different procedures: the typical titrimetric method^45^ and a spectroscopic method. For titrimetric determination, CA was refluxed in an ethanol solution at 75% v/v (1:50 solid:liquid ratio) at 40 °C for 30 min. Then the solution was cooled to room temperature, and 40 mL of NaOH 0.5 M was added. The reaction was allowed to stir for 72 h with mild agitation. This step is known as the saponification step, in which NaOH reacts with the acetate group producing CH_3_COONa proportionally to the amount of acetyl groups present in the cellulose. Unreacted NaOH is back-titrated with 0.5 M HCl and the DA is calculated using eqs 1 and 2

1 $$\mathrm{DA}=\frac{162\;A}{4300-\left(42\;A\right)}$$

2 $$A=\frac{\left(blank-sample\;titer\right)mL\times acid\;molarity\times 0.043\times 100}{sample\;weight\;\left(g\right)}$$

where *A* = percent acetyl.

The spectroscopic method is based on the ratio of the integrated areas of the absorption FTIR bands at 1736 cm^–1^ (C=O stretching of acetyl groups) and 1030 cm^–1^ (C–O stretching of the cellulose backbone).^46^ Peak deconvolution was performed by using OMNIC software to accurately calculate the peak areas.

*Films preparation* CA-based films were prepared using a casting technique based on the procedure reported by Gonçalves et al.,^47^ in which CA powder was dissolved in acetone at a 1:10 (w/v) ratio and stirred for 12 h to obtain a film-forming solution, and 10% (w/v) of glycerol was added as a plasticizer, incorporated at the beginning of the dissolution step to ensure better integration into the polymer matrix.

*Characterization* UV–vis absorption measurements were carried out at room temperature using a Varian Cary 100 Scan spectrophotometer (Middelburg, Netherlands) after baseline correction. Measurements were performed on plasticized and nonplasticized CA film samples (1 cm × 2 cm). Films were fixed on a cuvette spacer and positioned perpendicular to the incident light beam. Film opacity was calculated according to eq 3, where absorbance at 600 nm (representative of visible light) was normalized by film thickness.^48,49^

3 $$\mathrm{Opacity}=\frac{A_{600nm}}{\mathrm{Thickness}\;\left(\mathrm{mm}\right)}$$

FTIR analyses were conducted using a Thermo Scientific Nicolet iS50 FT-IR spectrometer equipped with an ATR accessory and OMNIC software. Spectra were recorded in the range of 4000–600 cm^–1^, with a resolution of 4 cm^–1^ and 32 scans per sample. A new background spectrum was acquired before each measurement.

Thermal properties of the polymers extracted from the waste biomass and of the CA-based film were investigated by Differential Scanning Calorimetry (DSC) and Thermogravimetric Analysis (TGA). DSC measurements were performed using a Discovery DSC 250 instrument (TA Instruments). Extracted lignin and cellulose samples were sealed in hermetic aluminum pans and heated at a rate of 10 °C/min up to 400 °C, under a nitrogen atmosphere (flow rate: 50 mL/min). For unplasticized and plasticized CA films, heat–cool–heat cycles were carried out to determine the glass transition temperatures (*T*_g_). TGA was performed by using a Mettler Toledo TG-DSC 1 Star System. Analyses were conducted under a nitrogen atmosphere (50 mL/min), heating from room temperature to 900 °C at 10 °C/min.

*Compostability tests* Compostability tests were conducted on cellulose acetate films plasticized with 10% glycerol (CA-10%GLY). For comparison, films of commercial cellulose diacetate plasticized with 10% glycerol (CCA-10%GLY) were synthesized to assess the degradation behavior of films derived from ultrapure polymer (CCA) versus those obtained from biomass-extracted cellulose (CA). Low-density polyethylene (LDPE) film was used as a negative control,^50,51^ while laboratory-grade filter paper (67 g/m^2^) served as the positive control. Samples were placed in direct contact with soil inside LDPE mesh nets (1.0–1.5 mm mesh size), which were also filled with soil to facilitate weekly sample retrieval. This setup allowed for easy recovery of the residual sample at each time point. Composting was performed in a 50 × 40 cm box containing 20 L of commercial soil (GEOVITAL Garden Service). The compost was refreshed weekly by adding a mixture of vegetable waste, homogenized, and manually mixed into the soil after the removal of test samples, to ensure adequate oxygenation and moisture. Each week, the samples were recovered upon removal from the bag, adhering soil was gently shaken off and further removed manually or with a soft brush to avoid affecting the integrity of the film, and the samples were weighed and buried again following compost refreshment. When possible, physical dimensions were also recorded. Environmental temperature and relative humidity were monitored throughout the experiment. The weight loss (in milligrams) of each sample was determined relative to its initial mass, averaged across replicates, and reported as a percentage. Standard errors were calculated for each time point. All tests were performed in duplicate. The procedure represents a disintegration test inspired by EN 13432:2000, which defines the requirements for packaging recoverable through composting and biodegradation. While the full EN 13432 protocol involves multiple assessments (biodegradation via CO_2_ evolution, disintegration by sieving, ecotoxicity, and heavy metal analysis), in this study only the disintegration criterion was addressed, under home-composting-like conditions. The objective was not to claim full compliance with EN 13432, but rather to comparatively evaluate the disintegration behavior of the CA-10%GLY film, its commercial analog (CCA-10%GLY), and the control samples (paper and LDPE).

## 3 Results

### 3.1 Effect of Green Pretreatments on Cellulose and Lignin Recovery

For an extraction process to be both efficient and economically sustainable, several key parameters must be considered, including the product yield, purity, and energy consumption. Equally important is the scalability of the technology, which must be evaluated from both economic and environmental perspectives to ensure its viability for industrial application. In this context, we evaluated the effectiveness of three green pretreatment strategies (ultrasound-assisted extraction (UAE), microwave-assisted extraction (MAE), and autoclave-assisted extraction (AAE)) all of which have been reported in the literature as scalable and environmentally friendly approaches.^29^ These methods were investigated with the aim of enhancing the separation and recovery of cellulose and lignin from the lignocellulosic biomass. Cellulose is a homopolymer of glucose units, while lignin is a complex polyaromatic polymer formed by radical polymerization of coniferyl, sinapyl, and *p*-coumaryl alcohols.^52,53^ Lignin’s amorphous structure, high molecular weight, and reactivity make it difficult to isolate in its native form, often leading to underutilization.^54,55^ Moreover, lignin acts as a structural barrier in the plant matrix, hindering the efficient extraction of cellulose and hemicellulose.^56–58^ The primary objective of pretreatment is to disrupt the complex structure of lignocellulose by cleaving ester and ether bonds, thereby facilitating the disassembly of the main components. Notably, hemicellulose is particularly challenging to remove due to its strong association with lignin; hence, an effective pretreatment is crucial for its efficient separation.

Our study employed these pretreatment methods as a necessary preliminary step to facilitate the effective extraction of cellulose and lignin by the organosolv method, ensuring sufficient separation of the biomass components while maintaining an acceptable balance among processing time, reagent consumption, and product quality. We carried out organosolv extraction of biomass previously treated with UAE, MAE, and AAE methods, and without any pretreatment, to evaluate the effectiveness of this step in enhancing the fractionation of lignocellulosic biomass.

Ultrasound-assisted pretreatment (UAE) is an effective green method for disrupting the lignocellulosic biomass. Ultrasonic cavitation generates the formation and violent collapse of microbubbles in the liquid medium, producing intense shear forces and localized high temperature and pressure gradients. These conditions disrupt the ordered structure of plant fibers, increasing cell wall permeability and causing the breakdown of hydrogen bonds, facilitating the release of intracellular components and thereby enhancing cellulose accessibility. UAE operates at mild bulk temperatures, requires minimal chemical addition, and reduces overall energy consumption, aligning with green chemistry goals.^59^ MAE is considered a green and efficient method for the disruption of lignocellulosic biomass. Microwaves generate rapid and uniform heating by directly interacting with polar molecules such as water, which enhances the internal temperature and pressure within the biomass structure. This effect leads to the weakening of hydrogen bonds and promotes the hydrolysis of hemicellulose, partial depolymerization of lignin, and the improved accessibility of cellulose. Unlike conventional heating, microwave irradiation enables energy-efficient processing with reduced reaction times, lower solvent usage, and minimal chemical input, following the principles of green chemistry.^60^ The mechanism of AAE involves the combined effect of the temperature, pressure, and saturated steam, which promotes hydrolysis and softens the biomass matrix. Upon depressurization due to the opening of the vent valve at the end of the treatment, rapid expansion and sudden evaporation of the water that has penetrated inside the matrix occur, causing physical disruption of cell walls. This enhances polymer separation, similar to steam explosion, but under milder conditions. Furthermore, the saturated steam atmosphere produced autoclaving, would also produce degradation phenomena that mainly affect the hemicellulose and increase with longer exposure times.^61,62^

The two biopolymers obtained after the three different pretreatments, cellulose and lignin, were analyzed by FTIR. The purity of cellulose was estimated by calculating the lignin index (LI) indicating the contamination of cellulose by lignin residues and calculated as the ratio of the absorbance of a characteristic lignin band to that of cellulose (C=C stretching of aromatic rings of lignin, at 1513 cm^–1^, and C–O stretching in glycosidic ether bonds for cellulose, at 1030 cm^–1^). The purity of lignin was estimated by the polysaccharide index (PI), indicating the polysaccharide contamination of lignin by cellulose and hemicellulose, and calculated as the ratio of the absorbance of the characteristic band of cellulose (1030 cm^–1^) to that of lignin (1513 cm^–1^). Table 2 reports the extraction yields (calculated as the ratio of dry product mass to that of the starting dry biomass) of cellulose and lignin pretreated with UAE, MAE, and AAE followed by organosolv extraction compared to the yields obtained without any pretreatment. LI calculated for cellulose, and PI calculated for lignin are also reported.

**Table 2:** Yields and FTIR-Derived Indices of Cellulose and Lignin Fractions Obtained after Different Pretreatments (Ultrasound-Assisted Extraction, Autoclave-Assisted Extraction, and Microwave-Assisted Extraction)^a^

	Cellulose		Lignin	
Green Treament	Yield %	LI	Yield %	PI
**None**	87.6 ± 0.8	0.27 ± 0.01	8 ± 1	2.99 ± 0.01
**UAE – 1 h**	78 ± 3	0.26 ± 0.01	6.2 ± 0.8	1.69 ± 0.01
**UAE – 2 h**	87.8 ± 0.5	0.27 ± 0.01	7 ± 1	1.36 ± 0.01
UAE – 3 h	86 ± 1	0.27 ± 0.01	7 ± 2	1.00 ± 0.01
**MAE**	85 ± 5	0.26 ± 0.01	5.1 ± 0.6	1.41 ± 0.01
**AAE**	73 ± 9	0.27 ± 0.01	7 ± 2	0.87 ± 0.05

a Lignin Index (LI) = $A_{1513{\mathrm{cm}}^{-1}}/A_{1030cm^{-1}}$ represents the lignin contamination in cellulose sample; Polysaccharide Index (PI) = $A_{1030{\mathrm{cm}}^{-1}}/A_{1513cm^{-1}}$ represents polysaccharides contamination in lignin sample.

Without pretreating the biomass, the organosolv extraction yielded 87.6 ± 0.8% of cellulose that showed lignin contamination after spectroscopical analysis by FTIR (Figure S1). The LI was calculated as 0.27, which corresponds to approximately 10% of lignin with respect to cellulose. A percentage yield of 8 ± 1% of lignin was recovered by organosolv extraction without pretreatment, showing a high polysaccharide contamination, as can be observed in the FTIR spectrum (Figure S2), where bands assigned to polysaccharides are clearly visible, and as can be deduced by a PI value of 3.

In the UAE, sonication was applied for 1, 2, and 3 h. After 1 h, yields were slightly lower than those obtained from untreated biomass (cellulose: 78 ± 3% vs 87.6 ± 0.8%; lignin: 6.2 ± 0.8% vs 8 ± 1%). While the contamination of lignin in cellulose is quite unchanged with respect to the untreated sample, the lignin purity was enhanced (PI: 1.69 vs 3.00). After 2 h, the extraction efficiency of cellulose increased, reaching values comparable to the control (cellulose: 87.8 ± 0.5%), with stable lignin contamination, while the extraction yield for lignin slightly increased with respect to 1 h treatment (7 ± 1% vs 6.2 ± 0.8), also showing purity improvement (PI decreased from 1.69 to 1.36). After 3 h ultrasound application, only an improvement in lignin purity was observed (PI: 1.00), suggested by a further reduction in polysaccharide contamination, while yields and cellulose purity were quite unchanged. Prolonged exposure can lead to depolymerization of the polysaccharide fraction.^37^ Moreover, ultrasound can induce the formation of reactive oxygen species capable of oxidizing biomass.^63^ This could explain the lower yield for lignin subjected to UAE, with respect to untreated sample, and its increased purity observed after 3 h irradiation, likely due to depolymerization and oxidative degradation of hemicellulose. The presence of hemicellulose is indeed evident in the FTIR spectrum of the untreated lignin, as indicated by the characteristic band around 1740 cm^–1^.

Microwave pretreatment decreased extraction efficiencies with respect to the untreated control for both cellulose (85 ± 5% vs 87.6 ± 0.8%) and lignin (5.1 ± 0.6% vs 8 ± 1%), and the resulting biopolymers showed significant contamination, even if the PI is quite lower than the one obtained without pretreating the sample.

Autoclave pretreatment followed by extraction resulted in 73 ± 9% and 7 ± 2% yields for cellulose and lignin respectively. The treatment did not improve cellulose purity but it led to the complete removal of polysaccharides from the lignin fraction, as confirmed by FTIR (Figure S1b) and a PI of 0.87. Our results show that 30 min autoclave treatment was sufficient to promote efficient polymer separation and yield high-purity lignin. Further cellulose purification was achieved with the bleaching step, described in Section 3.2.

Based on these findings, autoclave pretreatment was selected as the most effective method, in order to obtain both cellulose and lignin with a low degree of impurity. It substantially reduced lignin–polysaccharide contamination compared with direct extraction, supporting a more efficient fractionation of lignocellulosic biomass. This contamination is commonly attributed to the formation of lignin–carbohydrate complexes (LCCs), stabilized by glycosidic, benzyl ether, and γ-ester bonds. These linkages hinder the separation of lignin from hemicellulose and are a major challenge in biomass industrial processing.^64^ The ability of autoclave pretreatment to disrupt these associations (as evidenced by the disappearance of the band at 1730 cm^–1^, which can be referred to hemicellulose, Figure S1b) represents a valuable advancement, not only for improving lignin purity but also for enabling the valorization of nonedible biomass in sustainable material applications.^61,62,64,65^ The path followed by the purified lignin is the subject of a separate study, in which it was employed to synthesize sulfur-free, biodegradable nanoparticles loaded with a model drug for controlled drug release applications.^31^

### 3.2 Sustainable Purification of Cellulose through Optimized Bleaching Protocols

Since the cellulose extracted through autoclave-assisted and organosolv methods still contained approximately 10% lignin, it underwent a bleaching process to achieve further purification. In this step, the residual bound lignin was intentionally partially removed, with the aim of retaining a small amount of it in the product. This choice was guided both by environmental considerations, aiming to minimize chemical use and energy consumption throughout the synthesis pathway, and by the functional role that lignin can play in modulating material performance. Lignin, in fact, particularly when present in controlled amounts, has been reported to influence the mechanical behavior and thermal stability of polymer matrices. It acts as a natural functional additive, enhancing the properties of the final material and reducing the need for synthetic additives. Indeed, lignin should impart UV-shielding and antioxidant properties to the final Bioplastic film, making it suitable for sustainable packaging, especially for food.^17,18^

As described in Section 2, four different bleaching procedures were applied to the extracted cellulose. Some modifications to known procedures^41,42^ were introduced to enhance the removal of residual hemicellulose and lignin, while maintaining environmentally friendly conditions. Specifically, the reaction time of the bleaching treatment was slightly extended to improve its effectiveness.

In the FTIR spectra of cellulose obtained after the different bleaching procedures (Figure 2), the characteristic signals of cellulose can be identified in all the samples: ν­(C–O) stretching in primary and secondary alcohols at 1040–1060 cm^–1^, ν­(C–O–C) stretching of the glycosidic bridge at 1160 cm^–1^, C–O stretching in glycosidic ether bonds (∼1030 cm^–1^), and δ­(C–H) deformation of the β-glycosidic linkage at 897 cm^–1^. In addition, other signals were observed: the peak at 1737 cm^–1^ is a reliable indicator of hemicellulose, attributed to carbonyl ester vibrations (acetyl groups) in uronic acid ester groups;^64^ the signals at 1594, 1510, and 1262 cm^–1^, typical of lignin, are assigned to the vibration of aromatic C=C (phenylpropanoid rings), C=C stretching of aromatic rings, and C–O stretching of guaiacyl/syringyl units, respectively.

**Figure 2:**
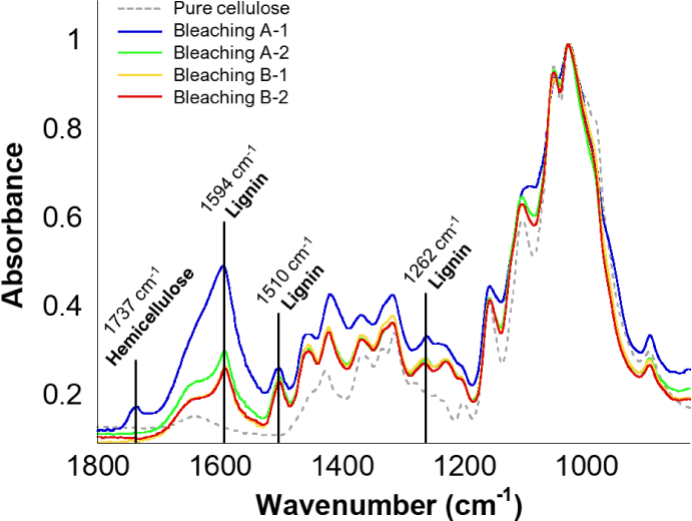
FTIR spectra of pure cellulose (dashed line) and bleached cellulose samples obtained from the different bleaching procedures: A-1 (4% NaOH, 30 min, blue line), A-2 (10% NaOH, 30 min, green line), B-1 (10% NaOH, 60 min, orange line), and B-2 (12% NaOH, 60 min, red line). Notably, the spectra of cellulose from procedures B-1 and B-2 are fully overlapped.

It can be observed that hemicellulose is removed in every bleaching procedure except procedure A-1 (7.5% hydrogen peroxide with 4% NaOH for 30 min at 100 °C), where a band at 1737 cm^–1^ is clearly visible (Figure 2, blue line). A higher concentration of NaOH (10%) successfully removed hemicellulose, but high residual lignin was still present. In contrast, procedure B, developed by combining previously reported protocols,^41,42^ employed 7.5% hydrogen peroxide with either 10% or 12% NaOH (procedures B-1 and B-2, respectively) for 60 min at 100 °C. This treatment resulted in cellulose free of hemicellulose and with significantly reduced lignin content, as shown by the lower peak intensities in the corresponding regions of the FTIR spectra. In particular, in procedures B-1 and B-2, the complete disappearance of the 1737 cm^–1^ FTIR band provides direct evidence for effective hemicellulose removal. Notably, increasing the NaOH concentration from 10% to 12% did not further improve cellulose purity, making protocol B-1 preferable due to lower reagent consumption.

The lignin content in the cellulose sample obtained from procedure B-1 was estimated by analyzing the ratio between the intensities of characteristic FTIR-ATR absorption bands associated with lignin and cellulose (1515 and 1030 cm^–1^). A calibration curve was generated by plotting this intensity ratio for five reference samples consisting of well-homogenized mixtures of pure cellulose and pure lignin at known concentrations. This approach enabled the quantification of residual lignin in the sample, which was determined to be 7.7 ± 1.8 wt % (Figure 3).

**Figure 3:**
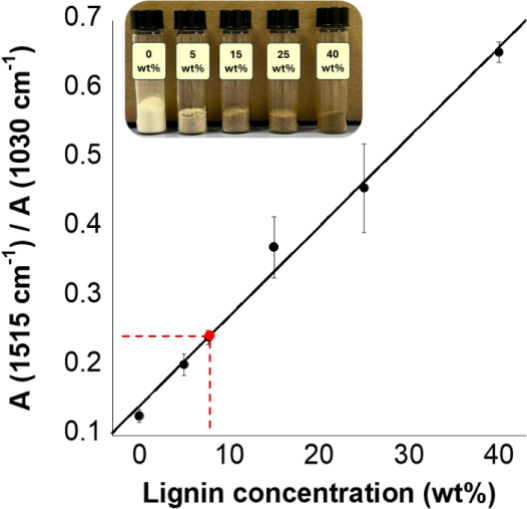
Calibration curve obtained by plotting the ratio of lignin-to-cellulose FTIR-ATR peak intensities as a function of lignin content (wt %) in reference samples composed of pure cellulose and organosolv lignin mixtures. The red point represents the experimental value calculated for the unknown sample, whose lignin content was interpolated using the regression equation. The correlation coefficient (*R* ^2^) of the calibration curve is 0.99547, indicating excellent linearity.

Notably, studies on cellulose-based nanopapers have shown that increasing the lignin content above ca. 5–10 wt % induces structural heterogeneity and phase separation, leading to the formation of voids and defects that compromise mechanical performances and overall material integrity.^66^ In our case, the observed lignin level remains below this threshold, helping to preserve material properties. Furthermore, compatibility is inherently enhanced in our system as lignin is not added exogenously but is already incorporated within the natural cellulose matrix, avoiding interfacial incompatibilities typical of external fillers.

The yield of cellulose obtained through the most effective bleaching protocol (B-1) was (46 ± 1)% calculated with respect to the initial biomass weight. This result demonstrates that optimizing cellulose extraction via autoclave treatment, combined with a modified bleaching process, is an effective strategy, as it enabled the recovery of a value-added secondary raw material from the lignocellulosic waste biomass. Figure 4 shows the visual appearance of the initial powdered biomass and the isolated components obtained, including cellulose-rich solid residue, cellulose bleached using procedure B-1, and lignin.

**Figure 4:**
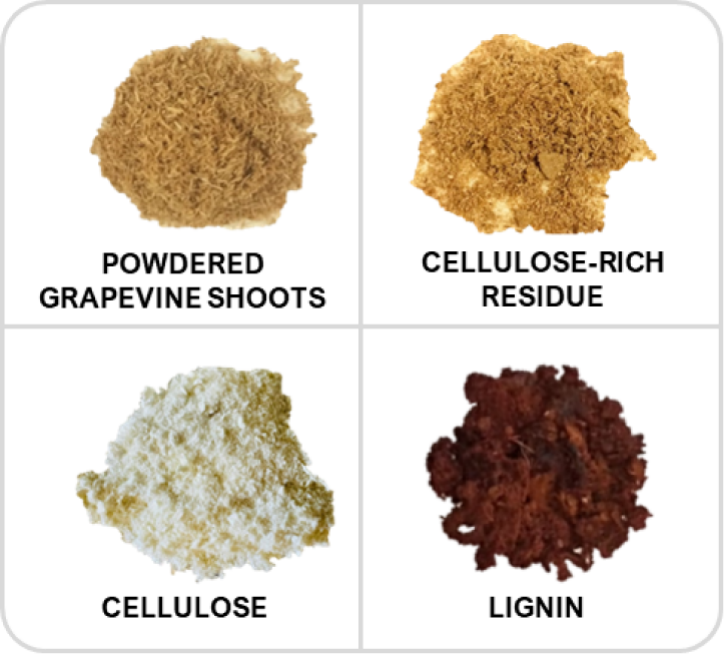
Visual appearance of the initial grapevine shoot biomass (powdered form) and extracted biopolymers: unbleached cellulose, bleached cellulose, and lignin.

### 3.3 Thermal Characterization of Bleached Cellulose and Lignin

Lignin and bleached cellulose, recovered through autoclave-assisted extraction and, in the case of cellulose, further purified using the most effective and environmentally sustainable bleaching protocol (B-1), were thermally characterized by DSC and TGA. The results shown in Figure 5 are in good agreement with the spectroscopic data.

**Figure 5:**
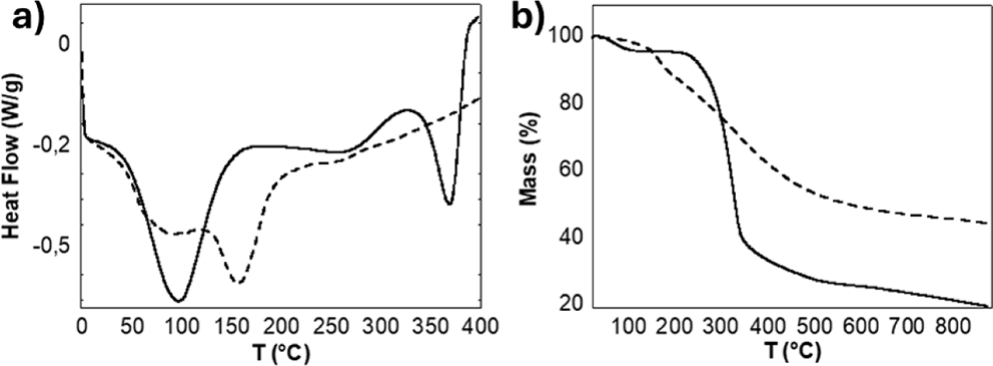
Thermal analysis of cellulose-3 (solid line) and lignin (dashed line), both derived from autoclave pretreatment and subsequent extraction a) DSC thermograms and b) TGA analysis.

In the DSC thermograms (Figure 5a), both cellulose and lignin exhibit broad endothermic signals below 120 °C, which are attributed to the release of physically adsorbed moisture. For cellulose (solid line), additional thermal events are observed: an endothermic peak between 200 and 280 °C, a weaker exothermic signal around 280–320 °C, and a pronounced endothermic peak centered at approximately 355 °C. The first event corresponds to the onset of cellulose thermal decomposition, involving the cleavage of glycosidic linkages, depolymerization, and the release of volatile degradation products. The exothermic event in the 280–320 °C range may be attributed to residual lignin, as supported by FTIR analysis, since lignin pyrolysis typically exhibits exothermic behavior over a broad temperature range. The third and more intense endothermic peak, occurring between 315 and 400 °C, is associated with the primary pyrolysis of cellulose. The distinction between these thermal events reflects the different mechanisms involved in the thermal degradation of cellulose and lignin. Cellulose decomposition is mainly governed by endothermic volatilization processes, whereas lignin degradation involves highly exothermic charring reactions.^67^ In fact, the DSC profile of lignin (dashed line) exhibits a clear exothermic trend, consistent with its complex, polyaromatic, and highly branched structure that undergoes slower and more heterogeneous thermal decomposition. Additionally, a broad endothermic signal is observed around 150 °C in the lignin thermogram, which can be attributed to the release of more strongly bound water, characteristic of lignin’s amorphous and hygroscopic nature.^68–71^

The TGA analysis (Figure 5b) further supports the DSC findings, confirming the thermal degradation profiles of both polymers. Cellulose exhibits a sharp decrease in mass (%) between 240 and 350 °C, corresponding to the rapid breakdown of its polysaccharide structure. In contrast, lignin degrades more gradually over a broader temperature range (150–500 °C), reflecting its complex molecular architecture, which includes aromatic rings, phenolic hydroxyl groups, and guaiacyl and syringyl units. These structural features, particularly the −OH-rich moieties, become increasingly thermally unstable, undergoing radical-driven rearrangements and bond cleavage at elevated temperatures. Guaiacyl-derived degradation products tend to form at lower temperatures, while syringyl-based fragments emerge at higher temperatures, contributing to the observed progressive mass loss. The thermogravimetric curves are consistent with literature data, including the residual mass at 900 °C: cellulose retains approximately 20 wt%, while lignin leaves around 45 wt%.^72,73^

### 3.4 Acetylation of Cellulose and Determination of Degree of Acetylation (DA)

Cellulose extracted after autoclave pretreatment and bleached using the B-1 protocol (cellulose-3) was then acetylated using acetic anhydride, combining experimental conditions described in the literature^43,44^ to which some modifications were applied to increase the sustainability of the entire process. The reaction time was selected with the aim of obtaining a diacetate. In fact, the literature reported that from 0.5 to 4 h of acetylation DS increases and then remains constant until 16 h, beyond this time range a triacetate product begins to be observed.^74^ In order to stop the reaction at the desired DS, and maintaining the reaction time as short as possible for energy saving, 4 h acetylation was used. The FTIR spectrum of the CA product indicates that the acetylation reaction has been successful (Figure S2) and characteristic CA peaks appears, at 1734 cm^–1^ is characteristic for acetyl bond and is proportional to the acetyl content.^74–77^ During the acetylation, since the −OH groups of cellulose are replaced by CH_3_COO- groups, the signal corresponding to the stretching vibration of the free OH groups (∼3420–3470 cm^–1^) decreases in intensity, while the stretching of the C=O ester group (1734 cm^–1^) increases.

The degree of acetylation (DA) was assessed using a spectroscopic approach based on FTIR analysis by calculating the ratio between the integrated absorption bands at 1734 cm^–1^ (C=O stretching of acetyl groups) and 1030 cm^–1^ (C–O stretching of the cellulose backbone), after peak deconvolution (Figure S3) obtaining a DA of 1.7. The resulting DA value was compared with that obtained through the more commonly used titration method, requiring 72 h of saponification with NaOH and subsequent back-titration with HCl (as described in Section 2). Using this method, the DA was found to be 2.2. While both methods provided consistent results, the FTIR-based approach offers clear sustainability advantages, being faster, requiring no chemical reagents and solvents, and significantly reducing energy consumption during analysis.^45^

### 3.5 CA-Based Film Preparation and Characterization

The synthesized cellulose acetate (CA) was subsequently formulated into a biobased film prototype for packaging applications, employing glycerol as a biobased and eco-friendly plasticizer. A glycerol content of 10 wt % was selected, as previous studies have demonstrated that this concentration provides enhanced flexibility and improved mechanical performance while maintaining the structural cohesion and biodegradability of CA films.^47^

A schematic representation of the plasticization of cellulose acetate with glycerol, increasing molecular mobility by intercalating between CA chains through hydrogen bonding interactions, and representative images of the films and a qualitative assessment of flexibility are presented in Figure 6.

**Figure 6:**
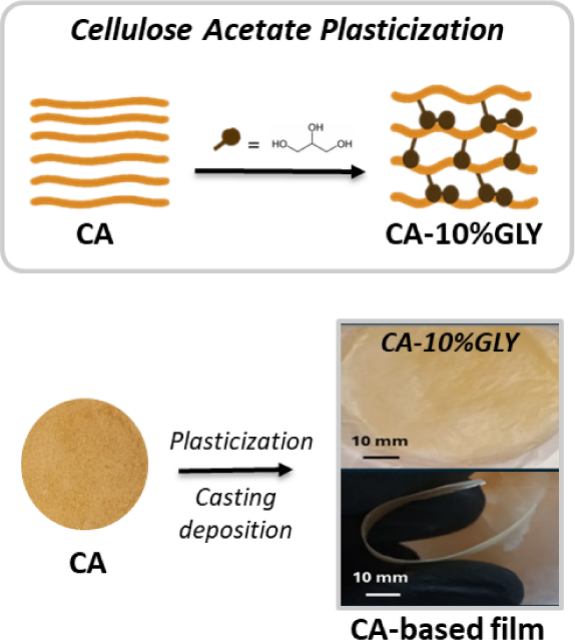
Schematic representation of the plasticization of cellulose acetate (CA) with 10 wt % glycerol (GLY), leading to increased molecular mobility. The plasticized CA (CA-10%GLY) was then processed by solvent casting to form flexible films. Visual appearance of the resulting CA-based films is shown on the right, highlighting the transparency and flexibility of the material.

The influence of glycerol on the chemical structure of the CA-based film was assessed by FTIR-ATR spectroscopy. The unplasticized sample showed the typical infrared spectrum of cellulose acetate (Figure S4) where the peak due to C=O stretching vibration of the acetyl group (∼1737 cm^–1^) is clearly visible. The absorbance of the ester carbonyl stretching band significantly decreased upon the addition of 10 wt % glycerol, dropping from 0.58 to 0.23. This reduction can be attributed to specific interactions between glycerol and the carbonyl groups of cellulose acetate,^78^ which likely affect the dipole moment of the C=O bond and reduce the effective number of IR-active ester groups. The addition of glycerol also produced the shift of specific signals: the peak at 898 cm^–1^, assigned to the β-1,4-glycosidic bond vibrations, shifted to 922 cm^–1^ in the plasticized sample, and a red shift in the O–H stretching band wavenumber occurred (from 3393 to 3340 cm^–1^), indicative of the formation of hydrogen bonds, which weaken the O–H bond, as part of the electron density of the −OH group is shared. As a result, the vibrational frequency decreases. These spectral changes strongly suggest the formation of specific interactions between glycerol and the cellulose acetate matrix, confirming that glycerol acts as a plasticizer by intercalating between cellulose acetate chains, thereby disrupting the native hydrogen-bonding network and promoting a reorganization of intermolecular interactions.

The thermal behavior of the cellulose acetate-based films shows expected changes with glycerol addition, as revealed by Differential Scanning Calorimetry (DSC). The glass transition temperature (*T*_g_) decreased from 126.49 to 121.32 °C (Figure 7) as a consequence of glycerol addition, consistently with the expected plasticizing effect, which increases free volume and chain mobility within the polymer matrix. This enhances flexibility in accordance with the free volume theory of plasticization^79^ and broadens the thermoplastic processability range of the material.^80,81^ Interestingly, the presence of residual lignin also influences the reduction in *T*_g_ because it acts as a plasticizer. In fact, the control film made from pure commercial cellulose acetate (CA) plasticized with the same amount of glycerol exhibits a higher glass transition temperature (125 °C) compared to that of the film containing lignin (Figure S5). Similar effect has been reported for acacia lignin-gelatin blended films using glycerol as plasticizer.^82^

**Figure 7:**
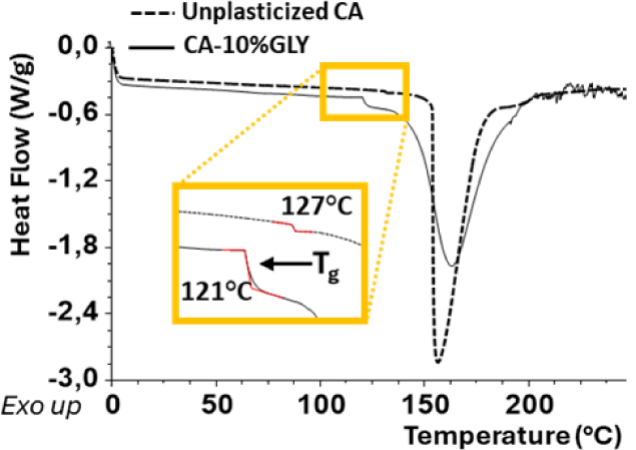
Differential scanning calorimetry thermograms of unplasticized cellulose acetate (CA) and CA plasticized with 10 wt % glycerol (CA-10%GLY). The plasticized sample shows a decrease in the glass transition temperature (*T*_g_) from 127 °C (unplasticized CA) to 121 °C, as highlighted in the magnified inset. This shift indicates enhanced polymer chain mobility due to glycerol incorporation.

The presence of lignin may disrupt the supramolecular organization of the cellulose acetate matrix by interacting with polymer chains and increasing the free volume between them. This perturbation of the polymer network is further enhanced by the inherently large structure of lignin, which hinders close chain packing and intermolecular interactions. Compared to lignins extracted by other methods, organosolv lignin exhibits a lower molecular weight, which facilitates its interaction with the polymer matrix, despite its inherently bulky structure. Additionally, it is likely that residual lignin underwent partial acetylation during the cellulose acetylation process. This chemical modification represents a further advantage, as the direct use of unmodified lignin in polymer systems is often limited by its poor compatibility and low reactivity. To address these drawbacks and enhance lignin’s applicability, several strategies have been described in the literature that exploit its high content of hydroxyl groups, allowing for various chemical modifications, such as acetylation, to render lignin more compatible and processable as a functional component in thermoplastic materials.^32^

*UV-shielding and transparency tests* An interesting and valuable feature of the synthesized film is its ability to shield ultraviolet (UV) radiation, which has been mainly attributed to the presence of residual lignin, which absorbs radiation in the UV range due to its polyphenolic structure. It was clearly demonstrated by comparing the UV–vis transmittance spectra (Figure 8) of the CA-based plasticized film with that of a film made from pure cellulose acetate plasticized with the same glycerol concentration (pure CA-based film). While the pure CA film allows partial transmission of UV light, our lignin-containing film shows complete blocking of UV-B and UV-C radiation and an excellent shielding effect also in the UV-A range, where the maximum value of transmittance is 17% at 400 nm. In general, the UV absorption of samples plasticized with glycerol is attributable to this plasticizer, which is known to have a shielding action from UV light.^83^ In this case, the effect is due both to the presence of glycerol (as a partial absorption of UV light is observed also in the pure CA sample) and to the presence of lignin.^84–86^ This UV-shielding capability is particularly important for packaging applications, especially in the food sector. Exposure to UV light can accelerate the degradation of food products, reducing shelf life. This provides a significant advantage for sustainable and active packaging solutions.

**Figure 8:**
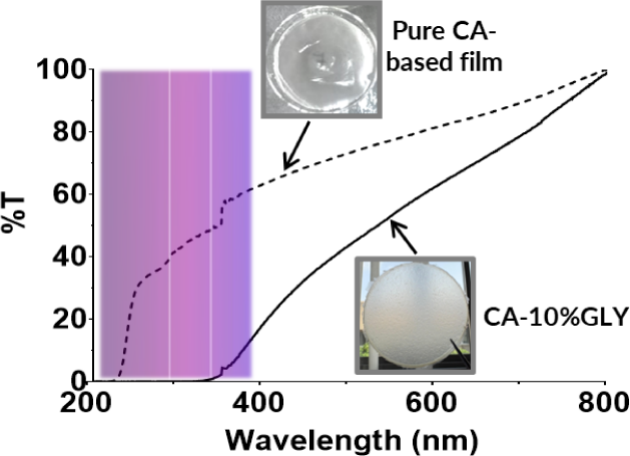
Transmittance UV–vis spectra and visual appearance of the CA-10%GLY sample and an analog film prepared using pure commercial CA.

Considering the visible range of the spectrum (400–800 nm), it was possible to calculate the film opacity by the ratio between the absorbance at 600 nm (representative of visible light) and the film thickness. According to the literature regarding polymeric films intended for food packaging, a film can be defined as “opaque” when *T* < 10%, “translucent” when the transmittance values are between 10% and 80%, and “transparent” when *T* > 80%.^87^ Our films fall within the definition of “translucent”, showing (63 ± 10)%T_600 nm_, while the pure CA-based film can be defined as “transparent”, with T_600 nm_ = (82 ± 2)%.

When comparing the opacity values reported in the literature for biobased polymeric films used in food packaging with those obtained for our control film (0.8 ± 0.2 mm^–1^) and the CA-10%GLY sample (1.2 ± 0.4 mm^–1^), our results align well with other cellulose acetate-based transparent films (opacity 1.0–1.5 mm^–1^), as well as with materials such as carboxymethyl cellulose and calcium alginate (opacity <1.5 mm^–1^).^87^ These findings confirm that the plasticized CA film developed in this study possesses optical properties suitable for food packaging applications, underlining its potential for commercial use in this sector.

*Compostabily test* The compostability of the films was assessed in accordance with the EN 13432:2000 standard, which provides guidelines for disintegration tests under controlled composting conditions. Plasticized cellulose acetate-based film (CA-10%GLY) was tested for compostability alongside an analog sample made on a pure commercial cellulose acetate sample (CCA-10%GLY), a positive control (filter paper), and a negative control (LDPE). The progression of film disintegration over time is illustrated in Figure 9, while the corresponding weight changes recorded throughout the composting period are summarized in the graph in Figure 10.

**Figure 9:**
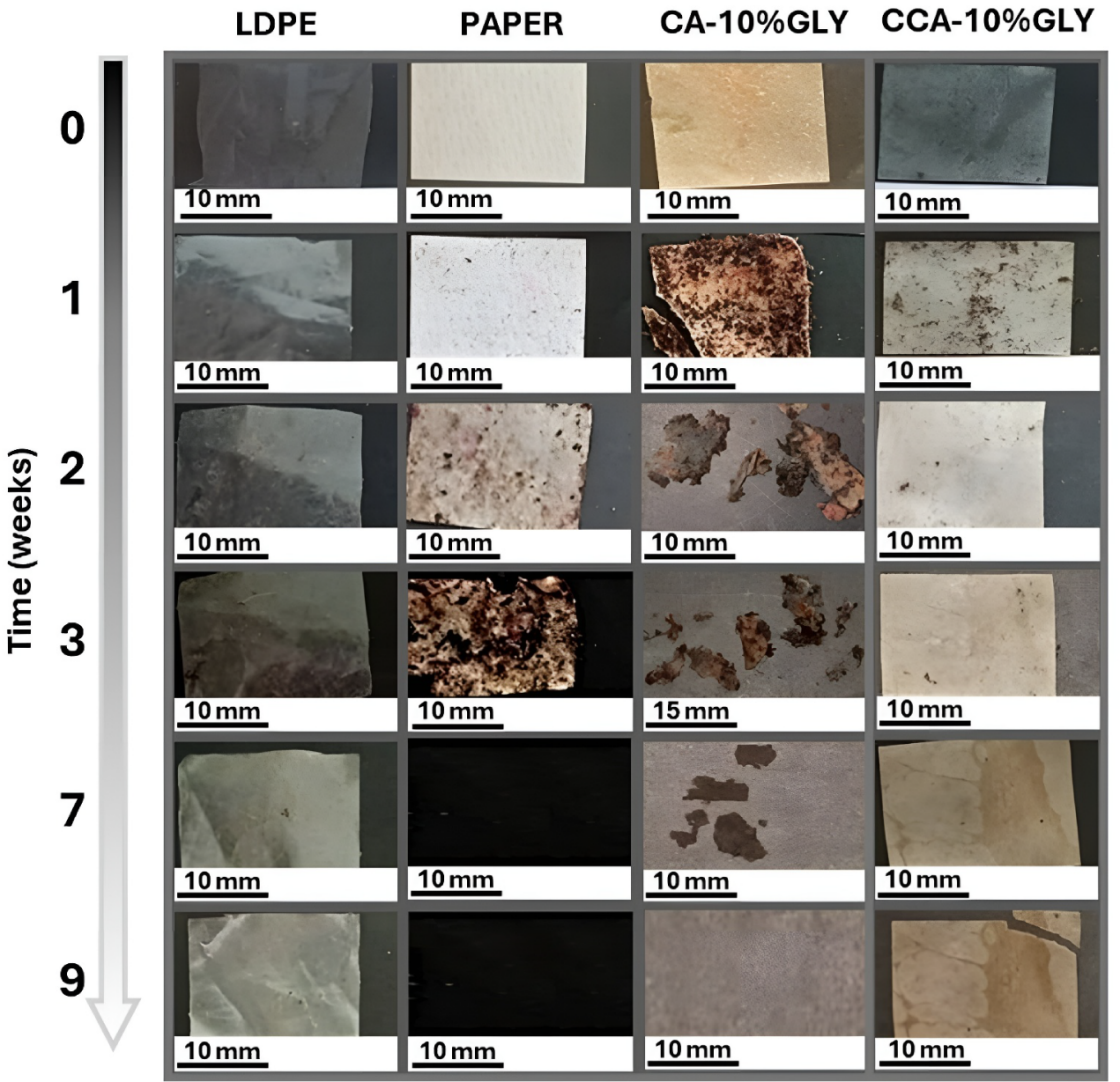
Visual progress of the degradation process in a home composter of negative (LDPE), positive (PAPER) control, and CA-10%GLY and CCA-10%GLY samples.

**Figure 10:**
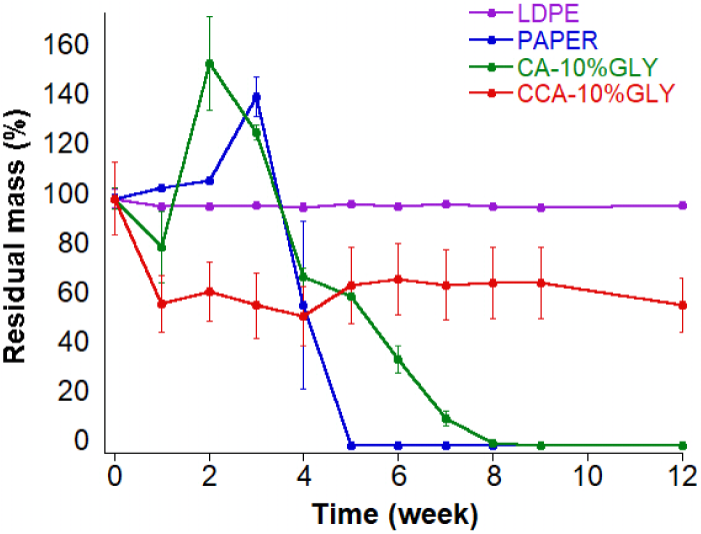
Residual mass (%) of tested materials over time during composting.

During composting, LDPE showed negligible weight change, confirming its nonbiodegradability, while filter paper exhibited typical behavior: initial weight gain due to moisture and soil adhesion, followed by drastic weight loss by week 4 and complete disintegration by week 5. Both paper and CA films developed reddish spots from week 2, likely due to foxing caused by interactions between fungi and transition metals under high-humidity conditions.^88^

CA-10%GLY fragmented within the first week (20% weight loss), with fungal colonization and yellowing evident. A transient weight increase at week 2 reflected water and soil retention. Subsequent rapid disintegration and microbial colonization led to ∼60% residual mass at week 4 and complete disintegration by week 8. The yellowing of CA, CCA, and paper samples is attributed to oxidative processes enhanced by compost conditions (humidity, acidity, and transition metals) which promote cellulose oxidation, introducing aldehyde and ketone groups that facilitate hydrolysis and increase acidity, ultimately affecting color and strength.^89^ The residual acidity in the synthesized CA likely accelerated degradation and foxing. The commercial CCA-10%GLY degraded more slowly, consistent with its higher substitution degree and lower residual acidity, which confer greater structural stability. Composting conditions (17–28 °C, 43–60% humidity) favored microbial growth, sustained by periodic addition of organic waste providing moisture, oxygen, and nutrients.^90^ These conditions promoted enzymatic deacetylation and chain scission by cellulolytic fungi and bacteria via endoglucanases, cellobiohydrolases, and β-d-glucosidases.^91–93^ Notably, the presence of residual lignin imparts a natural heterogeneity that can contribute to enhanced biodegradability without severely compromising the functional properties needed for transient uses.^94^ In addition, the biodegradation rate depends strongly on the degree of substitution (DS): lower DS (like our CA with DS ∼ 1.7–2.0) enables faster microbial hydrolysis, while higher DS (commercial CCA ∼ 2.0–2.2) slows degradation.^81,95,96^ Additional factors include molar mass, crystallinity, physical form, and environmental parameters (humidity, temperature, pH, oxygen, nutrients, microbial presence).^81,97^ The observed fragmentation and morphological changes confirm both physical disintegration and biological degradation. It is important to stress that the present test represents a disintegration study inspired by EN 13432 rather than a complete certification protocol. Nevertheless, the films fulfilled the visual and gravimetric disintegration criterion of the standard, showing complete disappearance within 8 weeks under ambient home-composting conditions. These conditions are less favorable than the industrial composting environment defined by EN 13432 (58 ± 2 °C, forced aeration, controlled humidity), and therefore, the rapid degradation observed here can be considered a conservative indicator of compostability. In addition to mass loss, clear signs of biological activity, including fungal colonization, foxing, and progressive yellowing, support the occurrence of active biodegradation rather than mere physical erosion. Literature reports complete disintegration of CA with DS ∼ 1.7 within 7 days under composting, while higher DS requires longer.^96^ Our films showed similar fragmentation times but slower full degradation (8 weeks), likely due to differences in experimental conditions and additives. Plasticizers significantly influence biodegradability; hydrophilic glycerol enhances water uptake, accelerating hydrolysis and microbial attack, supported by early fungal growth observations.^81,98^ Overall, the faster degradation of CA-10%GLY compared to CCA-10%GLY can be explained by the combined effect of its lower degree of substitution and the presence of residual acidity, which together increase the susceptibility to enzymatic hydrolysis and microbial attack. This highlights the importance of DS and chemical composition in tuning the compostability of cellulose acetate films for short-term biodegradable applications. Although molecular analyses (e.g., polymer weight, CO_2_ evolution) are needed for definitive biodegradation confirmation, our results demonstrate rapid and effective compostability of the synthesized CA films, consistent with literature and suitable for short-term biodegradable applications.

## 4 Conclusions

This work presents a comprehensive and eco-sustainable valorization strategy for grapevine pruning residues, an abundant and underutilized lignocellulosic waste from viticulture, by integrating green pretreatment, optimized extraction, and material conversion into high-value biopolymers. Within a zero-waste framework, cellulose and lignin were coextracted using mild autoclave conditions and low-catalyst organosolv treatment with green solvents, followed by a gentle bleaching step specifically designed to retain a controlled fraction of lignin within the cellulose matrix. Rather than being treated as an impurity, this residual lignin was strategically preserved for its functional properties, serving as an intrinsic additive that enhances the performance of the final material. To impart thermoplastic behavior, the recovered cellulose was acetylated to obtain cellulose diacetate (DS ≈ 1.7), which was processed into flexible, glycerol-plasticized Bioplastic films. The retained lignin played a pivotal role in endowing these films with additional functionality, particularly UV-shielding capability, a key feature for food packaging applications where photooxidation and discoloration must be avoided. Moreover, the films exhibited enhanced processability due to a reduced glass transition temperature, indicative of improved flexibility and thermal responsiveness imparted by plasticization. Most notably, the CA-10%GLY film achieved complete disintegration under home-composting conditions within 8 weeks, outperforming commercial cellulose acetate analogs. Although not a full UNI EN 13432:2000 compliance test, the results clearly show that lignin-containing cellulose acetate films undergo rapid disintegration and microbial attack, providing strong evidence of their suitability for short-term, compostable applications. The ability to degrade effectively even in milder, nonindustrial conditions highlights the sustainability advantage of these materials and their potential contribution to a circular bioeconomy framework.

This study underscores the dual role of lignocellulosic agricultural waste, as both a renewable feedstock and a source of naturally functional additives, and highlights the importance of process design that embraces circularity, multifunctionality, and low environmental impact. The proposed biorefinery model contributes to eco-sustainability by valorizing grapevine pruning waste, an abundant agricultural residue, through a green pretreatment that facilitates cellulose extraction without harmful reagents and avoids the chlorine- or sulfur-based chemicals typically used in conventional pulping. The intentional retention of lignin reduces purification steps, thereby lowering reagent and energy consumption while simultaneously providing intrinsic UV-shielding and antioxidant functionality to the resulting films without the need for synthetic additives. Moreover, the films exhibited rapid disintegration and clear microbial colonization under home-composting conditions, offering evidence of environmental degradation under mild, low-energy conditions. The strategy aligns with several principles of Green Chemistry and key directives of the European Circular Economy Action Plan (Regulation EU 2025/40; Waste Framework Directive), offering a scalable and sustainable route toward next-generation bioplastics and contributing meaningfully to global strategies for reducing plastic pollution.

## Supplementary Material



## Notes

Figure S1. FTIR spectra of cellulose and lignin extracted by organosolv method without pretreatment and after three biomass pretreatment; Figure S2. FTIR spectrum of cellulose acetate produced after 4h acetylation, overlapped to commercial cellulose acetate (CCA), used as a reference; Figure S3. Deconvolution analysis of FTIR spectra of synthesized CA; Figure S4. FTIR spectrum of cellulose acetate-based film, without plasticizer and with plasticizer; Figure S5. DSC thermogram of CCA-10%GLY showing the *T*_g_; Figure S6. Evidence of reddish foxing spots and strains after first week of composting in paper and CA-10%GLY; Figure S7. Composting of LDPE, paper, CA-10%GLY and CCA-10%GLY in parallel with the oscillations of temperature and the relative humidity (RH%) of the environment; Table S1. Weight variation of the tested materials during the soil burial test, expressed as percentage of the initial weight (% wt) and as weekly variation relative to the previous measurement (PDF)

## Abbreviations

CA: cellulose acetate
CCA: commercial cellulose acetate
DS: degree of substitution
DA: degree of acetylation
UAE: ultrasound-assisted extraction
AAE: autoclave-assisted extraction
MAE: microwave-assisted extraction

## References

[1] OECD. Global Plastics Outlook: economic Drivers, Environmental Impacts And Policy Options, OECD Publishing: Paris, 2022. https://www.oecd.org/en/events/2022/02/global-plastics-outlook-economic-drivers-environmental-impacts-and-policy-options.html. (accessed 16 April 2025).

[2] United Nations Environment Programme. Food Waste Index Report 2021, United Nations Environment Programme: Nairobi, 2021. https://www.unep.org/resources/report/unep-food-waste-index-report-2021. (accessed 22 March 2025).

[3] Circle Economy. The circularity gap report 2023, Circle Economy: Amsterdam, 2023, pp. 1–64. https://www.circularity-gap.world/2023#download. (accessed 11 April 2025).

[4] United Nations Environment Programme. Global Waste Management Outlook 2024: Beyond an age of waste – Turning rubbish into a resource, United Nations Environment Programme: Nairobi, 2024. https://wedocs.unep.org/handle/20.500.11822/44939;jsessionid=75C71C29455AE6C87F3C554ECE3C1F38. (accessed 11 April 2025).

[5] Korol J., Hejna A., Burchart-Korol D., Wachowicz J. (2020). Comparative
Analysis of Carbon, Ecological, and Water Footprints of Polypropylene-Based
Composites Filled with Cotton, Jute and Kenaf Fibers. Materials.

[6] Peças P., Carvalho H., Salman H., Leite M. (2018). Natural Fibre Composites
and Their Applications: A Review. J. Compos.
Sci..

[7] Khan A., Sapuan S. M., Yusuf J., Siddiqui V. U., Zainudin E. S., Zuhri M. Y. M., Baharuddin B. H. T., Ansari M. A., Rahman A. (2023). An examination
of cutting-edge developments in Bamboo-PLA composite research: A comprehensive
review. Renewable Sustainable Energy Rev..

[8] Drago E., Campardelli R., Lagazzo A., Firpo G., Perego P. (2023). Improvement
of Natural Polymeric Films Properties by Blend Formulation for Sustainable
Active Food Packaging. Polymers.

[9] Müller K., Zollfrank C., Schmid M. (2019). Natural Polymers from Biomass Resources
as Feedstocks for Thermoplastic Materials. Macromol.
Mater. Eng..

[10] Isikgor F. H., Remzi Becer C. (2015). Lignocellulosic biomass: A sustainable platform for
the production of bio-based chemicals and polymers. Polym. Chem..

[11] Rowell R. M. (2007). Challenges
in Biomass–Thermoplastic Composites. J. Polym. Environ..

[12] Ragauskas A. J., Beckham G. T., Biddy M. J., Chandra R., Chen F., Davis M. F., Davison B. H., Dixon R. A., Gilna P., Keller M. (2014). Lignin Valorization:
Improving Lignin Processing in
the Biorefinery. Science.

[13] Schutyser W., Renders T., Van den Bosch S., Koelewijn S. F., Beckham G. T., Sels B. F. (2018). Chemicals from Lignin:
An Interplay
of Lignocellulose Fractionation, Depolymerisation, and Upgrading. Chem. Soc. Rev..

[14] Del
Río J. C., Rencoret J., Prinsen P., Martínez Á. T., Ralph J., Gutiérrez A. (2012). Structural Characterization of Wheat
Straw Lignin as Revealed by Analytical Pyrolysis, 2DNMR, and Reductive
Cleavage Methods. J. Agric. Food Chem..

[15] Yao J., Karlsson M., Lawoko M., Odelius K., Hakkarainen M. (2023). Microwave-assisted
organosolv extraction for more native-like lignin and its application
as a property-enhancing filler in a light processable biobased resin. RSC Sustainability.

[16] Tardy B. L., Lizundia E., Guizani C., Hakkarainen M., Sipponen M. H. (2023). Prospects for the integration of lignin materials into
the circular economy. Mater. Today.

[17] Sadeghifar H., Ragauskas A. J. (2025). Lignin
as a Natural Antioxidant: Chemistry and Applications. Macromol.

[18] Wu X., Lian H., Xia C., Deng J., Li X., Zhang C. (2024). Mechanistic insights
and applications of lignin-based ultraviolet
shielding composites: A comprehensive review. Int. J. Biol. Macromol..

[19] Shrestha S., Fonoll X., Khanal S. K., Raskin L. (2017). Biological Strategies
for Enhanced Hydrolysis of Lignocellulosic Biomass during Anaerobic
Digestion: Current Status and Future Perspectives. Bioresour. Technol..

[20] Abo B. O., Gao M., Wang Y., Wu C., Ma H., Wang Q. (2019). Lignocellulosic
Biomass for Bioethanol: An Overview on Pretreatment, Hydrolysis and
Fermentation Processes. Rev. Environ. Health.

[21] Mujtaba M., Fernandes Fraceto L., Fazeli M., Mukherjee S., Savassa S. M., Araujo De Medeiros G., Do Espírito Santo
Pereira A., Mancini S. D., Lipponen J., Vilaplana F. (2023). Lignocellulosic
Biomass from Agricultural Waste to the Circular Economy: A Review
with Focus on Biofuels, Biocomposites and Bioplastics. J. Cleaner Prod..

[22] Güleç F., Parthiban A., Umenweke G. C., Musa U., Williams O., Mortezaei Y., Suk-Oh H., Lester E., Ogbaga C. C., Gunes B., Okolie J. A. (2024). Progress in Lignocellulosic Biomass
Valorization for Biofuels and Value-added Chemical Production in the
EU: A Focus on Thermochemical Conversion Processes. Biofuels, Bioprod. Biorefin..

[23] Wu D., Wei Z., Mohamed T. A., Zheng G., Qu F., Wang F., Zhao Y., Song C. (2022). Lignocellulose Biomass Bioconversion
during Composting: Mechanism of Action of Lignocellulase, Pretreatment
Methods and Future Perspectives. Chemosphere.

[24] Okolie J. A., Nanda S., Dalai A. K., Kozinski J. A. (2021). Chemistry and Specialty
Industrial Applications of Lignocellulosic Biomass. Waste Biomass Valorization.

[25] Putro J. N., Soetaredjo F. E., Lin S.-Y., Ju Y.-H., Ismadji S. (2016). Pretreatment
and Conversion of Lignocellulose Biomass into Valuable Chemicals. RSC Adv..

[26] Nanni A., Parisi M., Colonna M. (2021). Wine By-Products
as Raw Materials
for the Production of Biopolymers and of Natural Reinforcing Fillers:
A Critical Review. Polymers.

[27] Sánchez-García E., Martínez-Falcó J., Marco-Lajara B., Georgantzis N. (2024). Value Creation in the Wine Industry—a Bibliometric
Analysis. Eur. Food Res. Technol..

[28] Haldar D., Purkait M. K. (2021). A Review on the Environment-Friendly Emerging Techniques
for Pretreatment of Lignocellulosic Biomass: Mechanistic Insight and
Advancements. Chemosphere.

[29] Belwal T., Chemat F., Venskutonis P. R., Cravotto G., Jaiswal D. K., Bhatt I. D., Devkota H. P., Luo Z. (2020). Recent Advances in
Scaling-up of Non-Conventional Extraction Techniques: Learning from
Successes and Failures. Trends Anal. Chem..

[30] Tofani G., Jasiukaitytė-Grojzdek E., Grilc M., Likozar B. (2024). Organosolv
Biorefinery: Resource-Based Process Optimisation, Pilot Technology
Scale-up and Economics. Green Chem..

[31] Zadeh A. M., Gatto E., Lettieri R., Bokharaie H., Caravella A., D’Ottavi C., Di Bartolomeo E., Domenici F., Sima S., Correia A. (2025). Biomass-Derived
Lignin Nanoparticles for the Sustained Delivery of Vascular Endothelial
Growth Factor-C. Eur. J. Pharm. Biopharm..

[32] Duval A., Lawoko M. (2014). A review on lignin-based polymeric,
micro- and nano-structured
materials. React. Funct. Polym..

[33] Zijlstra D. S., Lahive C., Analbers C. A., Figueiredo M. B., Wang Z., Lancefield C., Deuss P. J. (2020). Mild organosolv
lignin extraction with alcohols: The importance of benzylic alkoxylation. ACS Sustainable Chem. Eng..

[34] Regulation - EU - 2025/40 - EN - EUR-Lex. https://eur-lex.europa.eu/eli/reg/2025/40/oj/eng. (accessed 24 April 2025).

[35] European Commission. Waste Framework Directive. https://environment.ec.europa.eu/topics/waste-and-recycling/waste-framework-directive_en (accessed 11 January 2025).

[36] Anastas, P. T. ; Warner, J. C. Green Chemistry: Theory and Practice; Oxford University Press: Oxford, NY, 2000.

[37] Abdullah M. A., Nazir M. S., Raza M. R., Wahjoedi B. A., Yussof A. W. (2016). Autoclave
and Ultra-Sonication Treatments of Oil Palm Empty Fruit Bunch Fibers
for Cellulose Extraction and Its Polypropylene Composite Properties. J. Cleaner Prod..

[38] Dranca F., Talón E., Vargas M., Oroian M. (2021). Microwave
vs. Conventional
Extraction of Pectin from Malus Domestica ‘Fălticeni’
Pomace and Its Potential Use in Hydrocolloid-Based Films. Food Hydrocolloids.

[39] Farid M. A. A., Ibrahim I., Lease J., Tsubota T., Andou Y. (2023). Effect of
Solvent and Acid Catalyst Selection on Lignin Recovery and Purity
in Autoclave-Assisted Organosolv Extraction. Bioresour. Technol. Rep..

[40] Xu F., Sun J.-X., Sun R., Fowler P., Baird M. S. (2006). Comparative
Study of Organosolv Lignins from Wheat Straw. Ind. Crops Prod..

[41] Gabriel T., Belete A., Syrowatka F., Neubert R. H. H., Gebre-Mariam T. (2020). Extraction
and Characterization of Celluloses from Various Plant Byproducts. Int. J. Biol. Macromol..

[42] Tezcan E., Atici O. G. (2017). Isolation of Cellulose
and Hemicellulose by Using Alkaline
Peroxide Treatment at Room Temperature from Wasted Fall Leaves. Nat. Eng. Sci..

[43] Djuned M. F., Asad M., Mohamad Ibrahim M. N., Wan Daud W. R. (2014). Synthesis and Characterization
of Cellulose Acetate from TCF Oil Palm Empty Fruit Bunch Pulp. BioResources.

[44] Egot M. P., Alguno A. C. (2018). Preparation and Characterization
of Cellulose Acetate
from Pineapple (Ananas Comosus) Leaves. KEM.

[45] Sodeinde K. O., Ojo A. M., Olusanya S. O., Ayanda O. S., Adeoye A. O., Dada T. M., Lawal O. S. (2021). Cellulose
Isolated from Delonixregia
Pods: Characterisation and Application in the Encapsulation of Vitamin
A. Ind. Crops Prod..

[46] Kramar A., Rodríguez Ortega I., González-Gaitano G., González-Benito J. (2023). Solution Casting of Cellulose Acetate
Films: Influence of Surface Substrate and Humidity on Wettability,
Morphology and Optical Properties. Cellulose.

[47] Gonçalves S. M., Dos Santos D. C., Motta J. F. G., Santos R. R. D., Chávez D. W. H., Melo N. R. D. (2019). Structure and Functional Properties of Cellulose Acetate
Films Incorporated with Glycerol. Carbohydr.
Polym..

[48] Chuenkaek T., Kobayashi T. (2024). Citrus Waste Upcycling toward Pectin Moisturizer Films
Plasticized with Glycerol and Polyethylene Glycol. ACS Sustainable Resour. Manage..

[49] Zhao J., Wang Y., Liu C. (2022). Film Transparency
and Opacity Measurements. Food Anal. Methods.

[50] Caravella A., Lettieri R., Vezza R., Gatto E. (2024). Aerobic Biodegradation
at a Seawater-Sediment Interface of a New Bioplastic 100% Based on
Natural Polymers. ACS Sustainable Resour. Manage..

[51] Ruggero F., Onderwater R. C. A., Carretti E., Roosa S., Benali S., Raquez J.-M., Gori R., Lubello C., Wattiez R. (2021). Degradation
of Film and Rigid Bioplastics During the Thermophilic Phase and the
Maturation Phase of Simulated Composting. J.
Polym. Environ..

[52] Yang J., Ching Y., Chuah C. (2019). Applications
of Lignocellulosic Fibers
and Lignin in Bioplastics: A Review. Polymers.

[53] Amthor J. S. (2003). Efficiency
of Lignin Biosynthesis: A Quantitative Analysis. Ann. Bot..

[54] Wang H., Pu Y., Ragauskas A., Yang B. (2019). From Lignin to Valuable Products–Strategies,
Challenges, and Prospects. Bioresour. Technol..

[55] Alam M. M., Greco A., Rajabimashhadi Z., Esposito Corcione C. (2024). Efficient
and Environmentally Friendly Techniques for Extracting Lignin from
Lignocellulose Biomass and Subsequent Uses: A Review. Cleaner Mater..

[56] Mankar A. R., Pandey A., Modak A., Pant K. K. (2021). Pretreatment of
Lignocellulosic Biomass: A Review on Recent Advances. Bioresour. Technol..

[57] Achinivu E. C., Mohan M., Choudhary H., Das L., Huang K., Magurudeniya H. D., Pidatala V. R., George A., Simmons B. A., Gladden J. M. (2021). A Predictive Toolset for the Identification of Effective
Lignocellulosic Pretreatment Solvents: A Case Study of Solvents Tailored
for Lignin Extraction. Green Chem..

[58] Singh N., Singhania R. R., Nigam P. S., Dong C.-D., Patel A. K., Puri M. (2022). Global Status
of Lignocellulosic Biorefinery: Challenges and Perspectives. Bioresour. Technol..

[59] Bussemaker M. J., Zhang D. (2013). Effect of Ultrasound on Lignocellulosic Biomass as a Pretreatment
for Biorefinery and Biofuel Applications. Ind.
Eng. Chem. Res..

[60] Bhatia S. K., Jagtap S. S., Bedekar A. A., Bhatia R. K., Patel A. K., Pant D., Banu J. R., Rao C. V., Kim Y.-G., Yang Y.-H. (2020). Recent developments
in pretreatment technologies on
lignocellulosic biomass: Effect of key parameters, technological improvements,
and challenges. Bioresour. Technol..

[61] Scherzinger M., Kaltschmitt M. (2019). Heat Induced
Pre-Treatment Technologies for Lignocellulosic
Biomass - A Comparison of Different Processes and Techniques. J. Ecol. Eng..

[62] Scherzinger M., Kulbeik T., Kaltschmitt M. (2020). Autoclave
Pre-Treatment of Green
Wastes – Effects of Temperature, Residence Time and Rotation
Speed on Fuel Properties. Fuel.

[63] Yasuda J., Yoshizawa S., Umemura S. (2016). Efficient Generation of Cavitation
Bubbles and Reactive Oxygen Species Using Triggered High-Intensity
Focused Ultrasound Sequence for Sonodynamic Treatment. Jpn. J. Appl. Phys..

[64] Wang W.-Y., Gao J.-H., Qin Z., Liu H.-M. (2022). Structural Variation
of Lignin-Carbohydrate Complexes (LCC) in Chinese Quince (Chaenomeles
Sinensis) Fruit as It Ripens. Int. J. Biol.
Macromol..

[65] Tarasov D., Leitch M., Fatehi P. (2018). Lignin–Carbohydrate Complexes:
Properties, Applications, Analyses, and Methods of Extraction: A Review. Biotechnol. Biofuels.

[66] Beluns S., Gaidukovs S., Platnieks O., Barkane A., Gaidukova G., Grase L., Nabels-Sneiders M., Kovalovs A., Thakur V. K. (2022). Effect
of Lignin Incorporation on the Properties of Cellulose Nanopapers. Carbohydr. Polym. Technol. Appl..

[67] Ball R., McIntosh A. C., Brindley J. (2004). Feedback processes
in cellulose thermal
decomposition: Implications for fire-retarding strategies and treatments. Combust. Theory Modell..

[68] López-Beceiro J., Díaz-Díaz A. M., Álvarez-García A., Tarrío-Saavedra J., Naya S., Artiaga R. (2021). The Complexity
of Lignin Thermal Degradation in the Isothermal Context. Processes.

[69] Yang H., Yan R., Chen H., Lee D. H., Zheng C. (2007). Characteristics of
Hemicellulose, Cellulose and Lignin Pyrolysis. Fuel.

[70] Lisperguer J., Perez P., Urizar S. (2009). Structure and thermal
properties
of lignins: Characterization by infrared spectroscopy and differential
scanning calorimetry. J. Chil. Chem. Soc..

[71] Yeo J. Y., Chin B. L. F., Tan J. K., Loh Y. S. (2019). Comparative Studies
on the Pyrolysis of Cellulose, Hemicellulose, and Lignin Based on
Combined Kinetics. J. Energy Inst..

[72] Kabir M. M., Alhaik M. Y., Aldajah S. H., Lau K. T., Wang H., Islam M. M. (2021). Effect of Hemp Fibre
Surface Treatment on the Fibre-Matrix
Interface and the Influence of Cellulose, Hemicellulose, and Lignin
Contents on Composite Strength Properties. Adv.
Mater. Sci. Eng..

[73] Kabir M. M., Wang H., Lau K. T., Cardona F. (2013). Effects of Chemical
Treatments on Hemp Fibre Structure. Appl. Surf.
Sci..

[74] Barud H. S., De Araújo Júnior A. M., Santos D. B., De Assunção R. M. N., Meireles C. S., Cerqueira D. A., Rodrigues Filho G., Ribeiro C. A., Messaddeq Y., Ribeiro S. J. L. (2008). Thermal Behavior
of Cellulose Acetate Produced from Homogeneous Acetylation of Bacterial
Cellulose. Thermochim. Acta.

[75] Candido R. G., Gonçalves A. R. (2016). Synthesis
of Cellulose Acetate and Carboxymethylcellulose
from Sugarcane Straw. Carbohydr. Polym..

[76] Das A. M., Ali A. A., Hazarika M. P. (2014). Synthesis
and Characterization of
Cellulose Acetate from Rice Husk: Eco-Friendly Condition. Carbohydr. Polym..

[77] Candido R. G., Godoy G. G., Gonçalves A. R. (2017). Characterization
and Application
of Cellulose Acetate Synthesized from Sugarcane Bagasse. Carbohydr. Polym..

[78] Friuli M., Grazioli C., Sattar N., Zia J., Del Sole R., Mergola L., Pal S., Licciulli A., Demitri C., Sannino A. (2025). Eco-Friendly Recovery
of Cellulose Acetate from Combusted Cigarette Filters and Reuse for
Membrane Fabrication. Waste Manage..

[79] Zahiruddin S. M. M., Othman S. H., Tawakkal I. S. M. A., Talib R. A. (2018). Mechanical and Thermal
Properties of Tapioca Starch Films Plasticized with Glycerol and Sorbitol. Food Res..

[80] Mekonnen T., Mussone P., Khalil H., Bressler D. (2013). Progress in
Bio-Based
Plastics and Plasticizing Modifications. J.
Mater. Chem. A.

[81] Bonifacio A., Bonetti L., Piantanida E., De Nardo L. (2023). Plasticizer Design
Strategies Enabling Advanced Applications of Cellulose Acetate. Eur. Polym. J..

[82] Aadil K., Barapatre A., Jha H. (2016). Synthesis and characterization of
Acacia lignin-gelatin film for its possible application in food packaging. Bioresour. Bioprocess..

[83] Teixeira S. C., Silva R. R. A., De Oliveira T. V., Stringheta P. C., Pinto M. R. M. R., Soares N. D. F. F. (2021). Glycerol and Triethyl Citrate Plasticizer
Effects on Molecular, Thermal, Mechanical, and Barrier Properties
of Cellulose Acetate Films. Food Biosci..

[84] Wang K., Xu F., Sun R. (2010). Molecular
Characteristics of Kraft-AQ Pulping Lignin
Fractionated by Sequential Organic Solvent Extraction. IJMS.

[85] Ammar M. (2017). Isolation
and Purification of Alfa Grass Kraft Lignin from Industrial Waste. Curr. Trends Biomed. Eng. Biosci..

[86] Sadeghifar H., Ragauskas A. (2020). Lignin as
a UV Light Blocker—A Review. Polymers.

[87] Guzman-Puyol S., Benítez J. J., Heredia-Guerrero J. A. (2022). Transparency of Polymeric Food Packaging
Materials. Food Res. Int..

[88] Choi S. (2007). Foxing on
Paper: A Literature Review. J. Am. Inst. Conserv..

[89] Area M. C., Cheradame H. (2011). Paper Aging and Degradation: Recent Findings and Research
Methods. BioRes.

[90] Environmental Protection Agency (EPA). Composting At Home. https://www.epa.gov/recycle/composting-home. (accessed 11 January 2025).

[91] Ghiya V. P., Dave V., Gross R. A., Mccarthy S. P. (1996). Biodegradability
of Cellulose Acetate Plasticized with Citrate Esters. J. Macromol. Sci., Part A.

[92] Buchanan C. M., Dorschel D., Gardner R. M., Komarek R. J., Matosky A. J., White A. W., Wood M. D. (1996). The Influence
of Degree of Substitution
on Blend Miscibility and Biodegradation of Cellulose Acetate Blends. J. Environ. Polym. Degrad..

[93] Komarek R. J., Gardner R. M., Buchanan C. M., Gedon S. (1993). Biodegradation of Radiolabeled
Cellulose Acetate and Cellulose Propionate. J. Appl. Polym. Sci..

[94] Huang M.-R., Li X.-G. (1998). Thermal Degradation
of Cellulose and Cellulose Esters. J. Appl.
Polym. Sci..

[95] Yadav N., Hakkarainen M. (2021). Degradable
or Not? Cellulose Acetate as a Model for
Complicated Interplay between Structure, Environment and Degradation. Chemosphere.

[96] Edgar K. J., Buchanan C. M., Debenham J. S., Rundquist P. A., Seiler B. D., Shelton M. C., Tindall D. (2001). Advances in Cellulose
Ester Performance and Application. Prog. Polym.
Sci..

[97] Nigam S., Das A. K., Matkawala F., Patidar M. K. (2022). An Insight Overview
of Bioplastics Produced from Cellulose Extracted from Plant Material,
Its Applications and Degradation. Environ. Sustainability.

[98] Nigam S., Das A. K., Patidar M. K. S. (2021). Characterization and Biodegradation
of Bioplastic Films Produced from Parthenium Hysterophorus by Incorporating
a Plasticizer (PEG600). Environ. Challenges.

