# Supplementary material for: Lignin-Containing Cellulose Acetate Films from Grapevine Waste: A Sustainable Path to Compostable Bioplastics
Source: ACS Sustain Chem Eng. 2025 Sep 16;13(38):16178–91. doi: 10.1021/acssuschemeng.5c07998 (PMC12486438; doi:10.1021/acssuschemeng.5c07998)
Supplement: Supplementary file 1 [file sc5c07998_si_001.pdf]

Supporting Information

Title: Lignin-Containing Cellulose Acetate Films from Grapevine Waste: A Sustainable Path to Compostable Bioplastics

Raffaella Lettieri [1,2]*, Alice Caravella [1], Giulia Quintarelli [1], Cadia D'Ottavi [1], Silvia Licoccia [1], Emanuela Gatto [1,2]*

[1] Department of Chemical Science and Technologies, University of Rome Tor Vergata, via della Ricerca Scientifica 1, 00133, Rome Italy.

[2] Splastica srl, spinoff of the University of Rome Tor Vergata, Via del Lavoro 13, Genzano di Roma 00045, Rome, Italy; operational headquarter in Via della Ricerca Scientifica 1, 00133, Rome Italy.

*Corresponding Authors: emanuela.gatto@uniroma2.it, raffaella.lettieri@uniroma2.it

Number of pages: 8
Number of figures: 7
Number of tables: 1

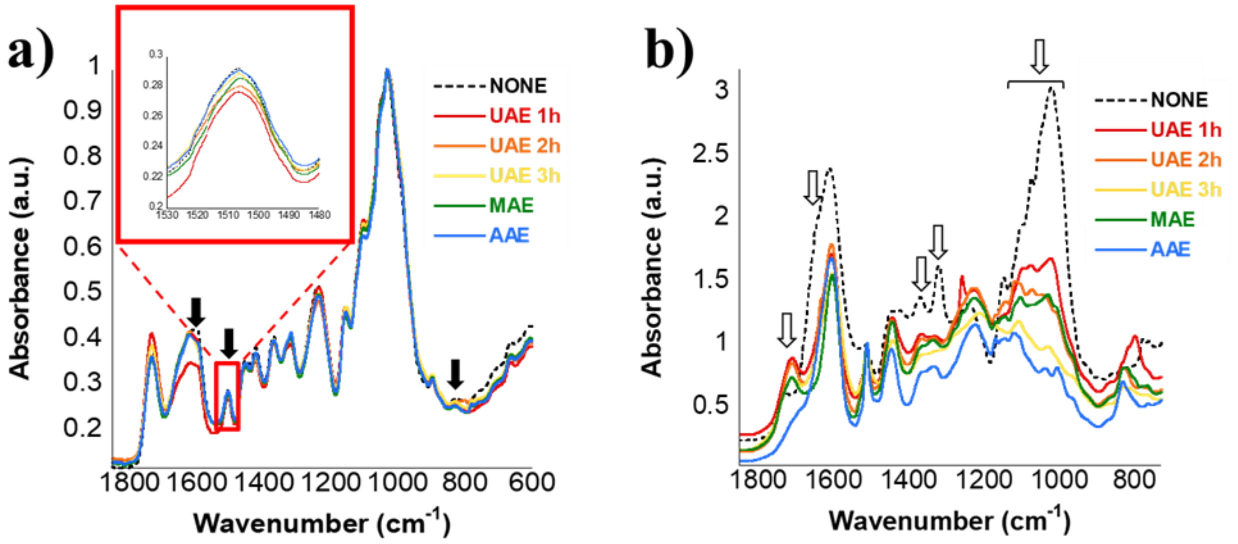

Figure S1. FTIR spectra of cellulose (a) and lignin (b) extracted by organosolv method without pretreatment and after three biomass pretreatment: ultrasound assisted extraction (UAE), autoclave assisted extraction (AAE), microwave assisted extraction (MAE).

In the spectra of cellulose (a) the presence of lignin residues is confirmed by the bands assigned to functional groups characteristic of lignin, mainly at 1506 and 1595 cm^{-1} due to the C=C stretching of aromatic rings, and at 830 cm^{-1} due to aromatic C-H bending, indicated by black arrows. In the spectra of lignin (b) the bands assigned to polysaccharides, indicated by white arrows, confirmed the polysaccharide contamination of lignin: C–H bending at 1375 cm^{-1}; C–H rocking, OH bending at 1335 cm^{-1}; band at 1030 cm^{-1} assigned to the stretching vibration of C–O bonds, with contributions from the glycosidic C–O–C ether linkage between glucose units; band at 1150–1160 cm^{-1} assigned to the asymmetric stretching vibration of C–O–C linkages, including the β(1→4) glycosidic bonds and the ring oxygen in the glucopyranose units; band at 1105 cm^{-1} corresponding to the C–O stretching vibration of secondary alcohol groups (C2 and C3 positions), typical of the hydroxyl functionalities in cellulose; the band at 1730-1740 cm^{-1} can be attributed to C=O stretching from acetyl groups or uronic acids, commonly found in hemicelluloses, particularly in xylans and glucomannans. The peak at ~1600 cm^{-1} is mainly referred to the C=C stretching vibrations of aromatic rings, a typical signature of lignin, but it can be noticed that in the non treated sample, the band also shows a contribution at ~1630 cm^{-1} for the presence of COO^{-} groups (e.g., from hemicelluloses containing uronic acids).

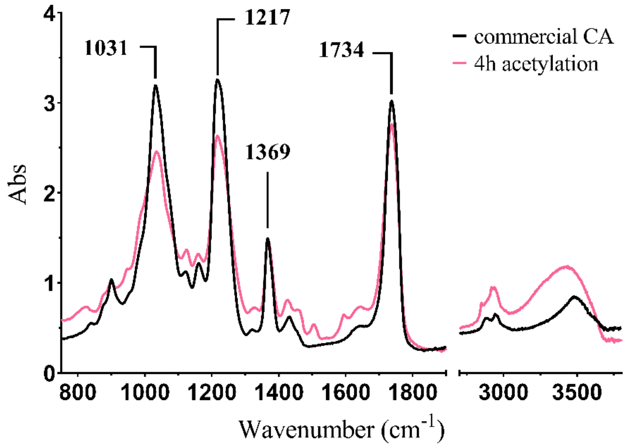

Figure S2. FTIR spectrum of cellulose acetate produced after 4h acetylation, overlapped to commercial cellulose acetate (CCA), used as a reference. Characteristic peaks are shown at 1734 cm^{-1}, corresponding to stretching vibration C=O of ester bond between acetyl group and cellulose, 1220 cm^{-1} corresponding to stretching C-O or -CO- in acetyl group, 1369 cm^{-1} corresponding to CH vibration, and 1031 cm^{-1} corresponding to vibration of O=C-O-CH_3 typical of sp^2 hybridization of cellulose acetate.

(a)

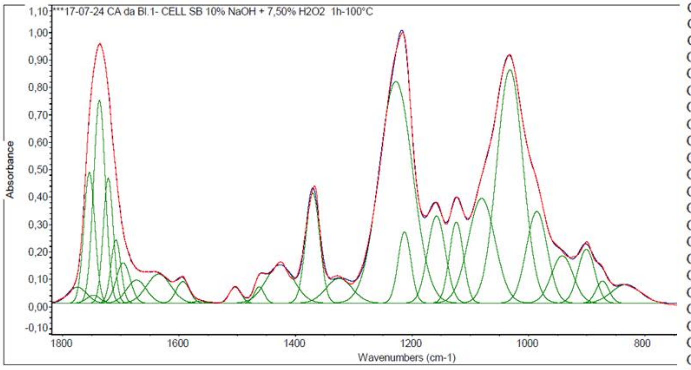

Peak Type	Center X	Height	FWHH	Other	Area
Gaussian	596,137	0,1302	30,731	0,000	4,2598
Gaussian	637,653	0,0236	35,184	0,000	0,8852
Gaussian	718,099	0,0000	19,296	0,000	0,0000
Gaussian	833,003	0,0680	57,187	0,000	4,1420
Gaussian	873,094	0,0806	25,948	0,000	2,2268
Gaussian	900,847	0,1969	33,710	0,000	7,0659
Gaussian	942,074	0,1733	43,577	0,000	8,0376
Gaussian	985,745	0,3349	42,074	0,000	14,9967
Gaussian	1031,965	0,8516	52,782	0,000	47,8472
Gaussian	1080,583	0,3825	54,065	0,000	22,0113
Gaussian	1123,908	0,2960	27,931	0,000	8,7997
Gaussian	1158,021	0,3186	35,619	0,000	12,0796
Gaussian	1212,953	0,2602	25,672	0,000	7,1100
Gaussian	1227,818	0,8091	67,261	0,000	57,9270
Gaussian	1325,211	0,0902	52,807	0,000	5,0694
Gaussian	1370,445	0,4016	27,801	0,000	11,8851
Gaussian	1426,712	0,1402	55,941	0,000	8,3469
Gaussian	1462,479	0,0596	19,622	0,000	1,2449
Gaussian	1503,755	0,0586	23,355	0,000	1,4577
Gaussian	1552,781	0,0012	22,900	0,000	0,0302
Gaussian	1563,557	0,0080	24,578	0,000	0,2101
Gaussian	1593,504	0,0796	26,578	0,000	2,2511
Gaussian	1634,960	0,1059	48,443	0,000	5,4633
Gaussian	1673,104	0,0842	39,597	0,000	3,5504
Gaussian	1695,934	0,1470	28,053	0,000	4,3894
Gaussian	1708,513	0,2314	19,872	0,000	4,8958
Gaussian	1721,636	0,4555	20,053	0,000	9,7225
Gaussian	1736,714	0,7401	23,579	0,000	18,5753
Gaussian	1747,231	0,0292	26,225	0,000	0,8149
Gaussian	1754,058	0,4767	22,069	0,000	11,1983
Gaussian	1774,794	0,0583	37,569	0,000	2,3305

(b)

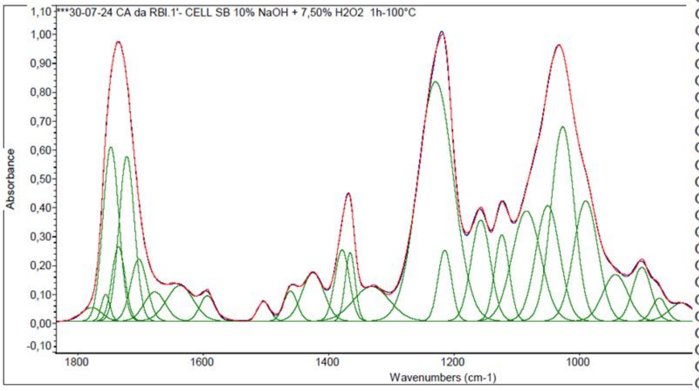

Peak Type	Center X	Height	FWHH	Other	Area
Gaussian	596,014	0,1519	32,018	0,000	5,1778
Gaussian	632,648	0,0375	31,712	0,000	1,2649
Gaussian	664,655	0,0215	34,893	0,000	0,7986
Gaussian	716,436	0,0021	23,078	0,000	0,0508
Gaussian	836,272	0,0629	47,291	0,000	3,1646
Gaussian	872,354	0,0795	26,440	0,000	2,2368
Gaussian	900,158	0,1866	34,320	0,000	6,8164
Gaussian	942,403	0,1613	46,567	0,000	7,9963
Gaussian	989,563	0,4164	45,152	0,000	20,0128
Gaussian	1026,497	0,6728	43,014	0,000	30,8047
Gaussian	1050,276	0,3997	44,570	0,000	18,9649
Gaussian	1084,287	0,3815	53,273	0,000	21,6318
Gaussian	1123,572	0,2986	26,945	0,000	8,5660
Gaussian	1157,302	0,3500	36,056	0,000	13,4327
Gaussian	1214,763	0,2460	25,028	0,000	6,5537
Gaussian	1229,298	0,8293	66,317	0,000	58,5407
Gaussian	1331,804	0,1195	67,470	0,000	8,5838
Gaussian	1365,733	0,2373	18,509	0,000	4,6757
Gaussian	1378,593	0,2465	26,952	0,000	7,0715
Gaussian	1424,985	0,1672	40,952	0,000	7,2894
Gaussian	1461,032	0,1043	25,186	0,000	2,7955
Gaussian	1503,668	0,0680	22,954	0,000	1,6610
Gaussian	1548,744	0,0032	23,783	0,000	0,0822
Gaussian	1593,707	0,0879	27,669	0,000	2,5900
Gaussian	1637,370	0,1229	49,788	0,000	6,5131
Gaussian	1677,753	0,1012	39,587	0,000	4,2655
Gaussian	1703,051	0,2138	30,491	0,000	6,9394
Gaussian	1722,336	0,5703	28,932	0,000	17,5632
Gaussian	1735,562	0,2579	24,344	0,000	6,6826
Gaussian	1747,663	0,6034	29,546	0,000	18,9785
Gaussian	1756,069	0,0926	15,848	0,000	1,5613
Gaussian	1778,303	0,0471	37,099	0,000	1,8586

Figure S3. Deconvolution analysis of FTIR spectra related to replicate 1 (a) and replicate 2 (b) of synthesized CA.

Deconvolution analyses were performed using OMNIC software and for all spectra an automatic baseline correction was done, followed by an automatic normalization using the function “normalize scale”. The set up for deconvolution were “Gaussian” for “find peak” option, “Low” for the “sensitivity”, “25” for “FWHM”, “0” as “noise target” and “Constant” as “baseline”. In figure S1 the deconvolution analysis results are shown.

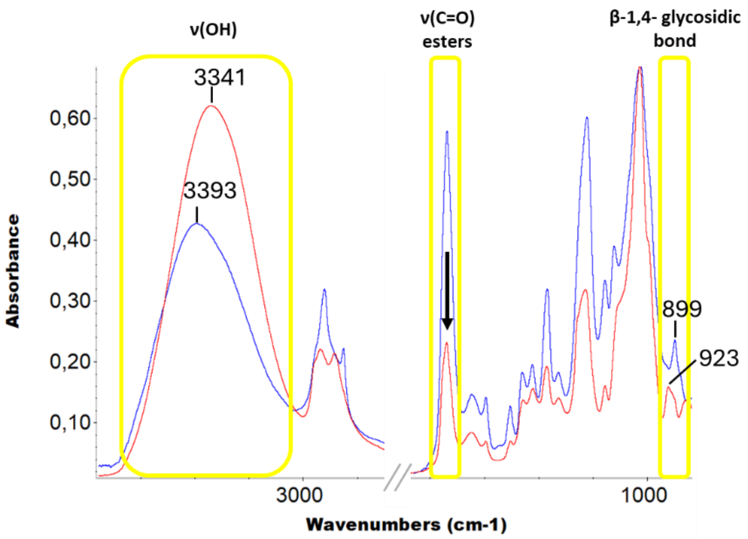

Figure S4. FTIR spectrum of cellulose acetate-based film, without plasticizer (blue line) and with plasticizer (CA-10%GLY, red line).

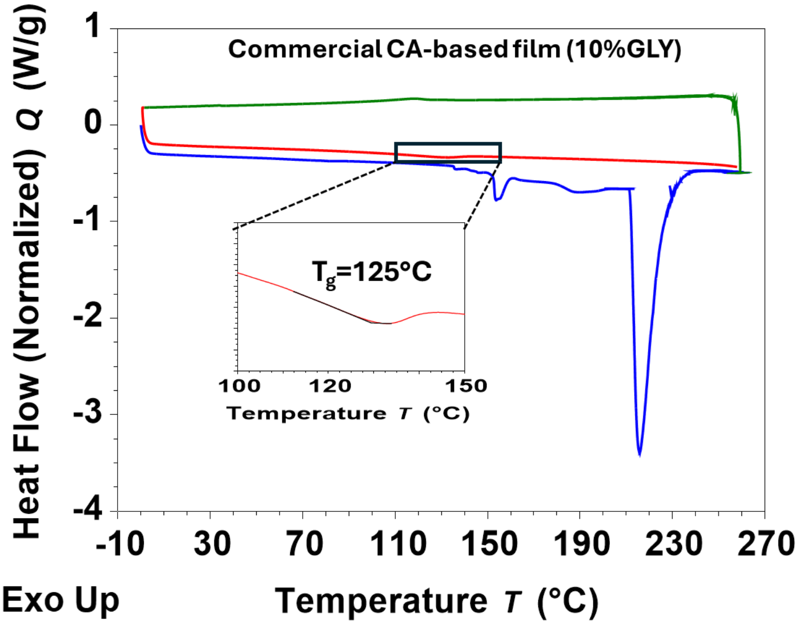

Figure S5. DSC thermogram of control film, made by commercial pure cellulose acetate and plasticized with 10% glycerol. The T_g value is higher than the corresponding film made by cellulose acetate containing lignin.

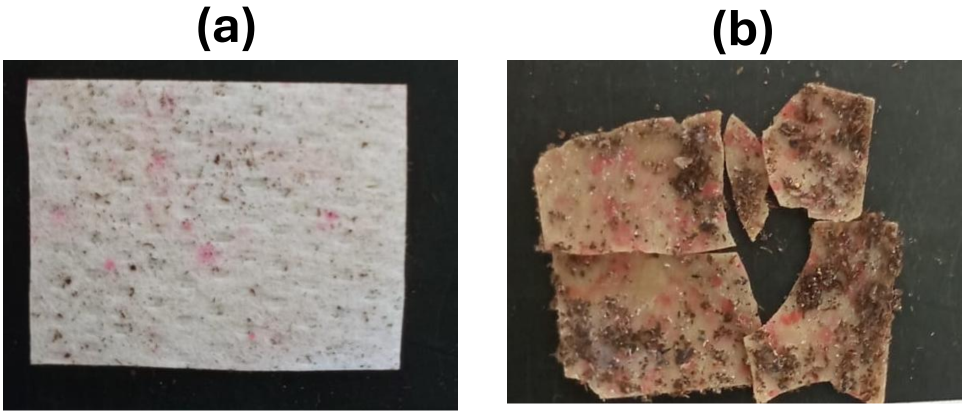

Figure S6. Evidence of reddish foxing spots and strains after first week of composting in paper (a) and CA-10%GLY (b).

Reddish dots are probably related to foxing process that could be related to transition metals contamination (iron, tin, copper, copper-mercury, copper-zinc, brass), microorganisms actions (fungi and bacteria), combinations of transition metals and fungi, oxidation, moisture condensation. (Choi, https://doi.org/10.1179/019713607806112378) In our case, foxing spots seems to be more related to a combination of metals and fungi action as compost is generally rich in iron, organic compounds and microorganisms that degrade organic waste. According to this mechanism, fungi produce acids in the cellulose, this acid reacts with ferrous salts (belonging to the soil or related to paper impurities) forming organic ferrous salts that with time decompose by oxidation producing iron oxide or hydroxide some of which has brown-reddish color. It's probable that these reactions occurs simultaneously in paper in contact with soil, producing reddish spots. In damp conditions a synergic reaction between fungi and metals is observed. In home composter, relative humidity was quietly high (40-60%), thus it is conceivable that high humidity in the soil has enhanced this foxing mechanism. On the other side, metals represent micronutrients essential for fungal growth and hyphae has a chelating ability for iron, copper and zinc.

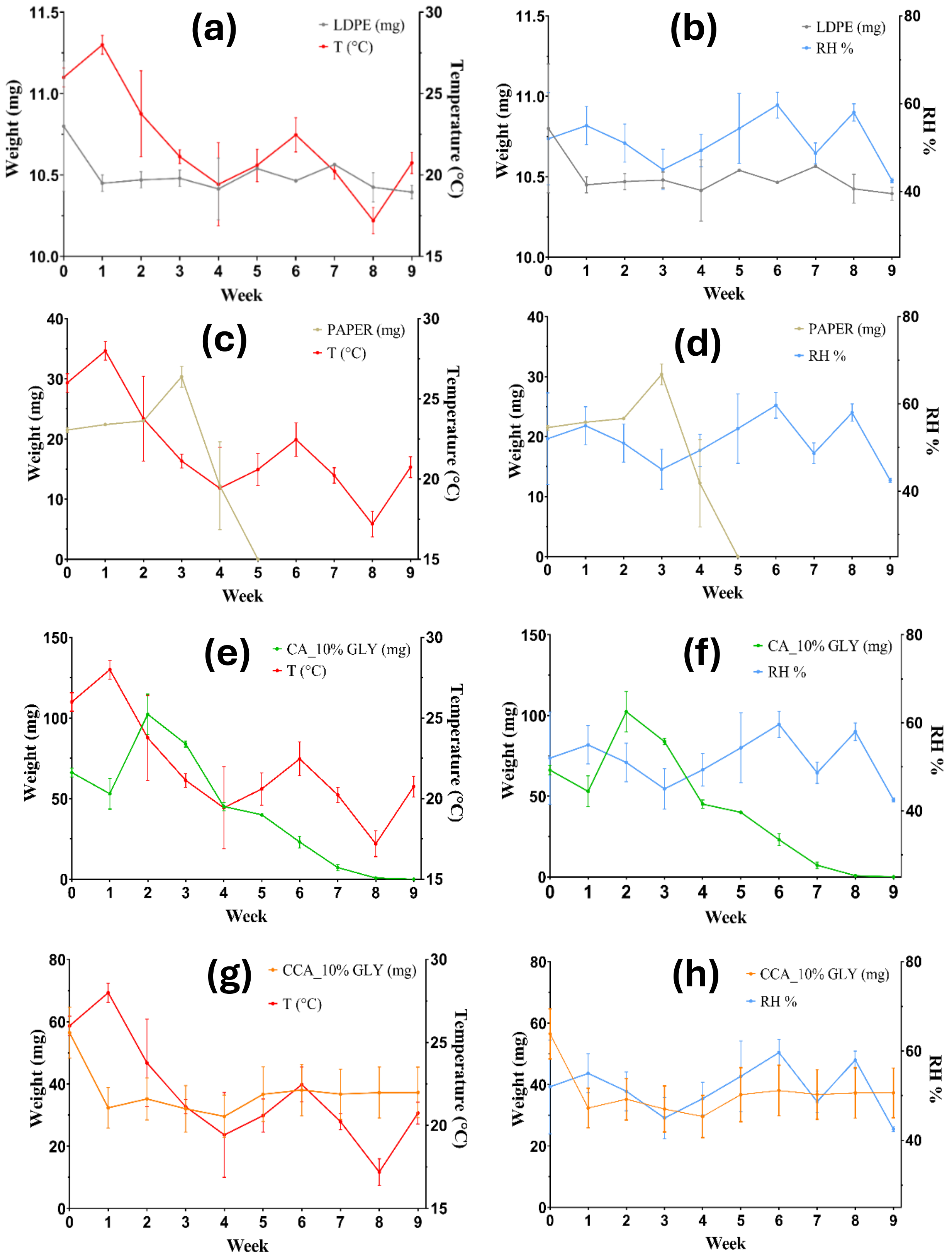

Figure S7. Composting of LDPE, paper, CA-10%GLY and CCA-10%GLY in parallel with the oscillations of temperature and the relative humidity (RH%) of the environment.

	LDPE		PAPER		CA_10% GLY		CCA_10% GLY	
	% wt	Δ wt%	% wt	Δ wt%	% wt	Δ wt%	% wt	Δ wt%
**WEEK 0**	100	-	100	-	100	-	100	-
**WEEK 1**	97	-3%	104	+4%	80	-20%	57	-43%
**WEEK 2**	97	-	107	+3%	155	+75%	62	+5%
**WEEK 3**	97	-	141	+34%	127	-28%	57	-5%
**WEEK 4**	96	-1%	57	-84%	68	-59%	52	-5%
**WEEK 5**	98	+2%	0	-57%	60	-8%	65	+13%
**WEEK 6**	97	-1%	0	-	35	-25%	67	+2%
**WEEK 7**	98	+1%	0	-	11	-24%	65	-2%
**WEEK 8**	97	-1%	0	-	1	-10%	66	+1%
**WEEK 9**	96	-1%	0	-	0	-1%	66	-

Table S1. Weight variation of the tested materials (LDPE, paper, cellulose acetate with 10 wt% glycerol (CA_10% GLY), commercial cellulose acetate with 10 wt% glycerol (CCA_10% GLY)) during the soil burial test. The values are expressed as percentage of the initial weight (% wt) and as weekly variation relative to the previous measurement (Δ wt%, highlighted in pink).
